# Supplementary material for: Evidence for gene essentiality in Leishmania using CRISPR
Source: PLoS One. 2024 Dec 30;19(12):e0316331. doi: 10.1371/journal.pone.0316331 (PMC11684651; doi:10.1371/journal.pone.0316331)

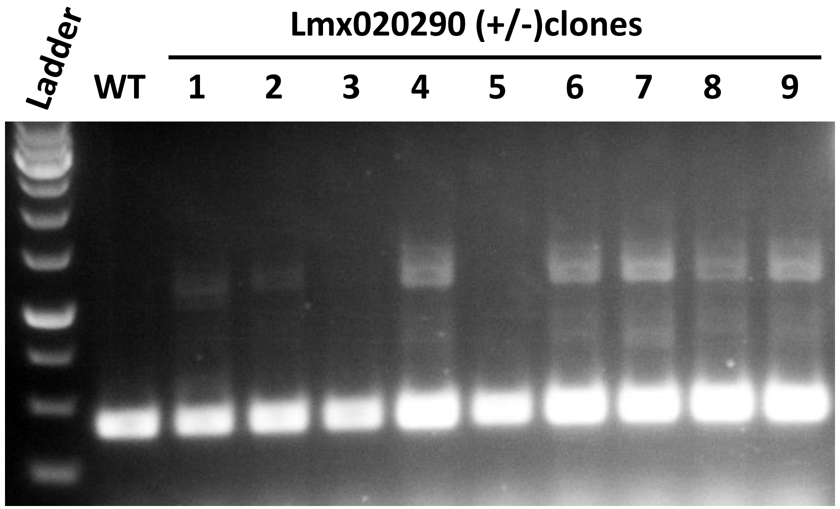
Lmx020290 (+/-)clones
Ladder
WT
1
2
3
4
5
6
7
8
9

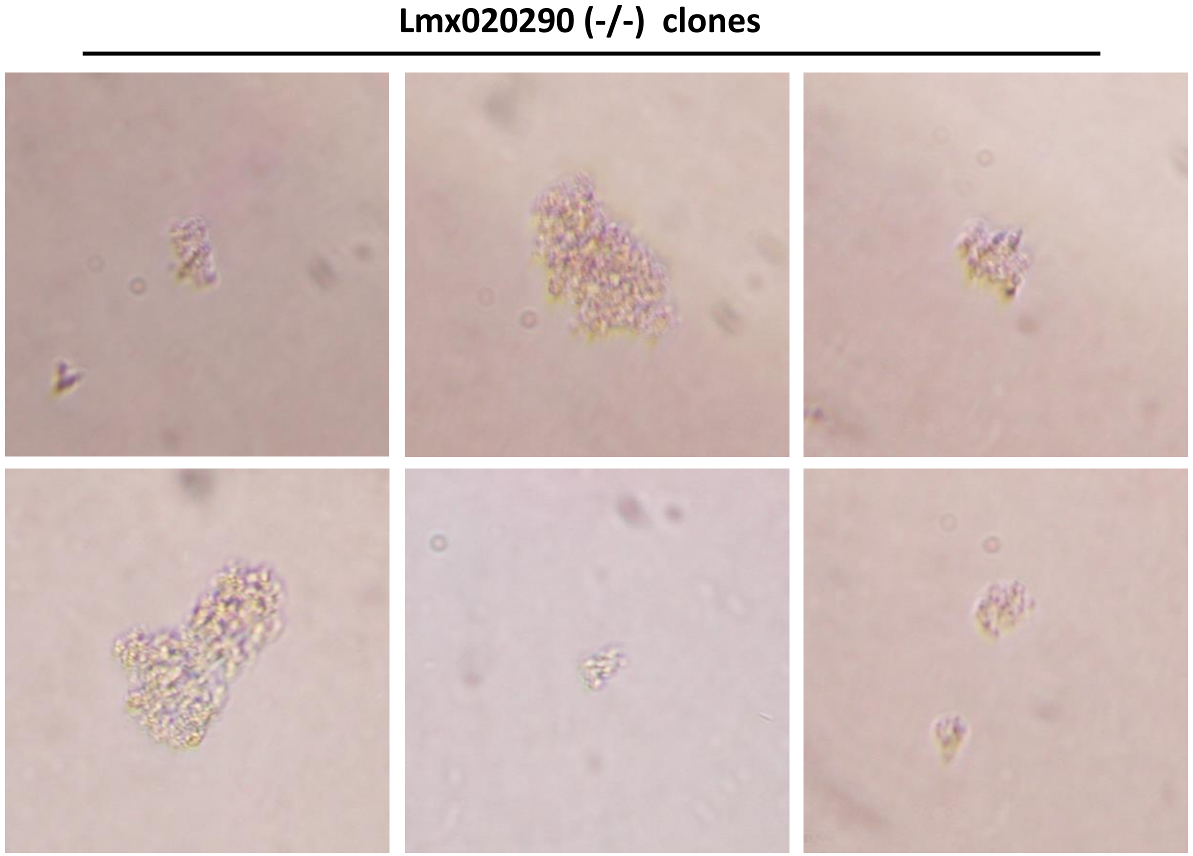
Lmx020290 (-/-) clones

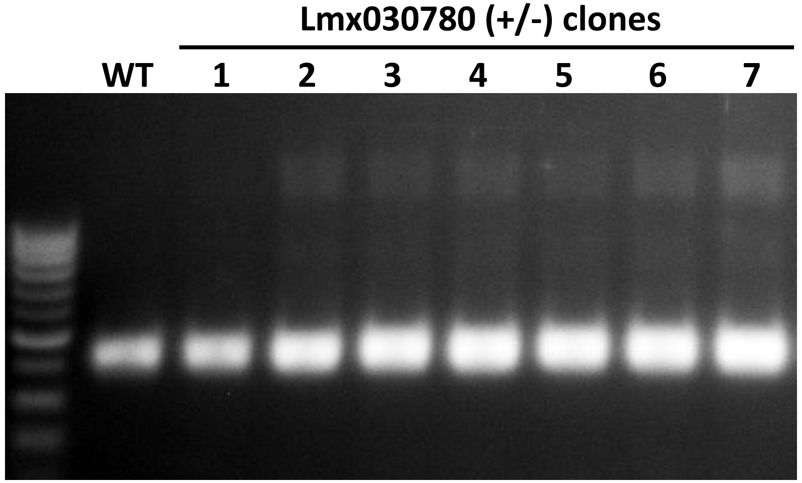
Lmx030780 (+/-) clones
WT
1
2
3
4
5
6
7

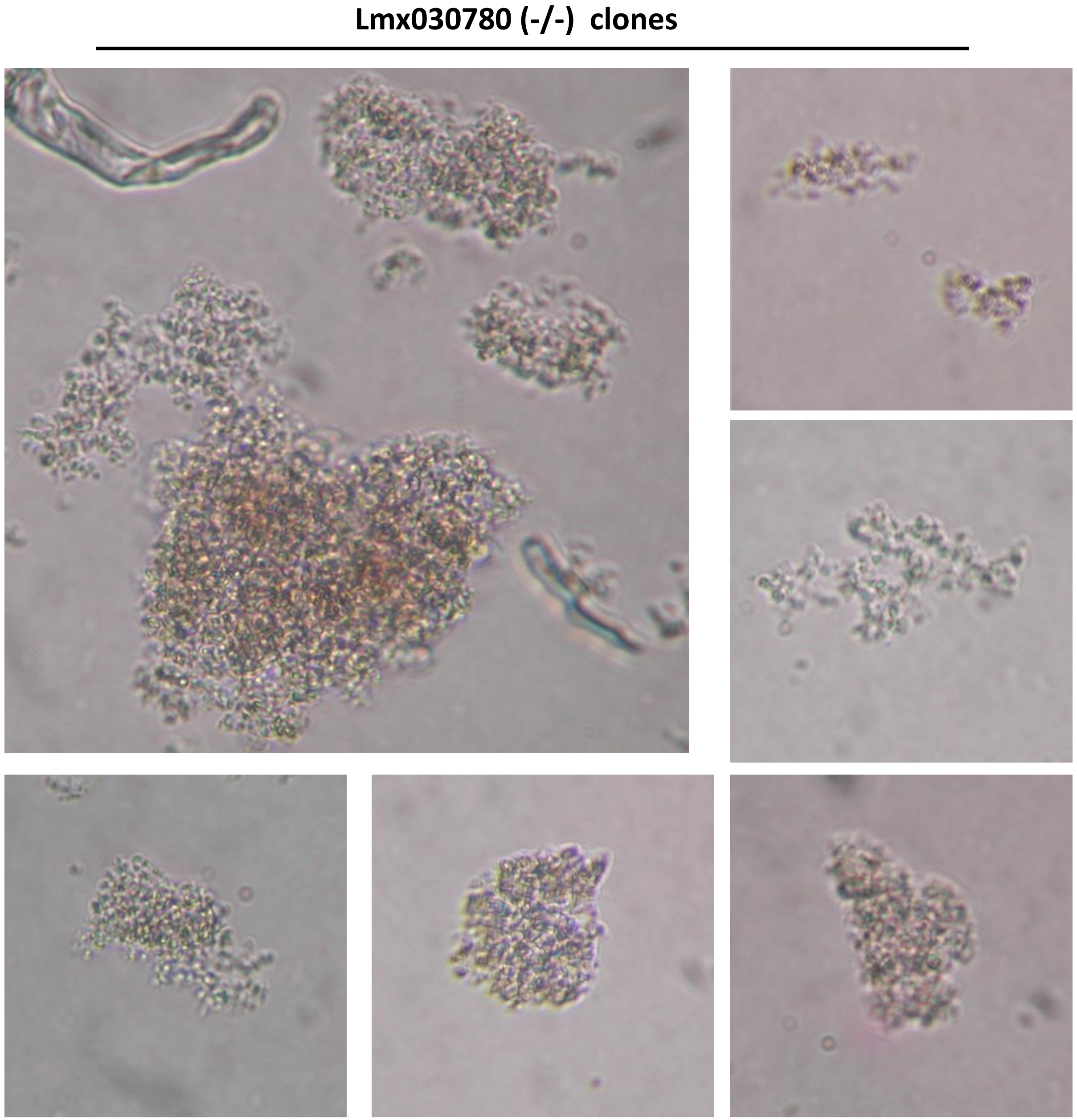
Lmx030780 (-/-) clones

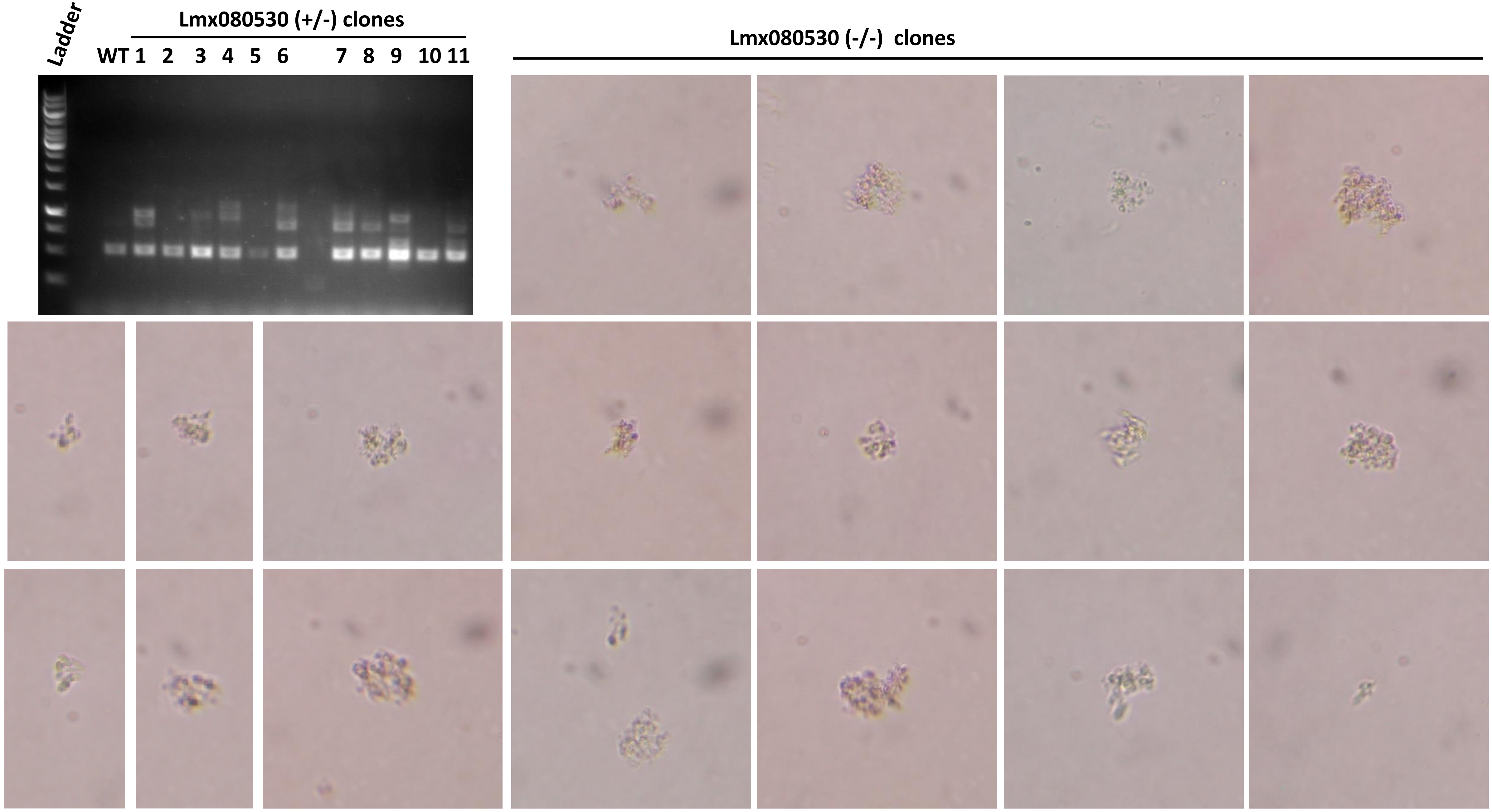

Ladder
Lmx080530 (+/-) clones
WT 1 2 3 4 5 6 7 8 9 10 11
Lmx080530 (-/-) clones

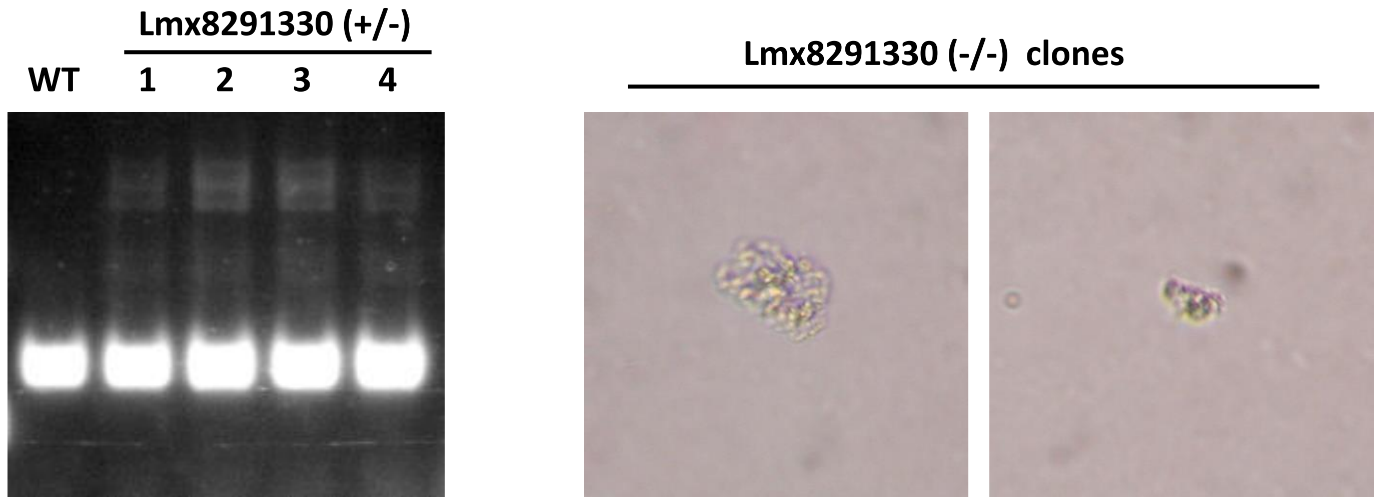
Lmx8291330 (+/-)
WT
1
2
3
4
Lmx8291330 (-/-) clones

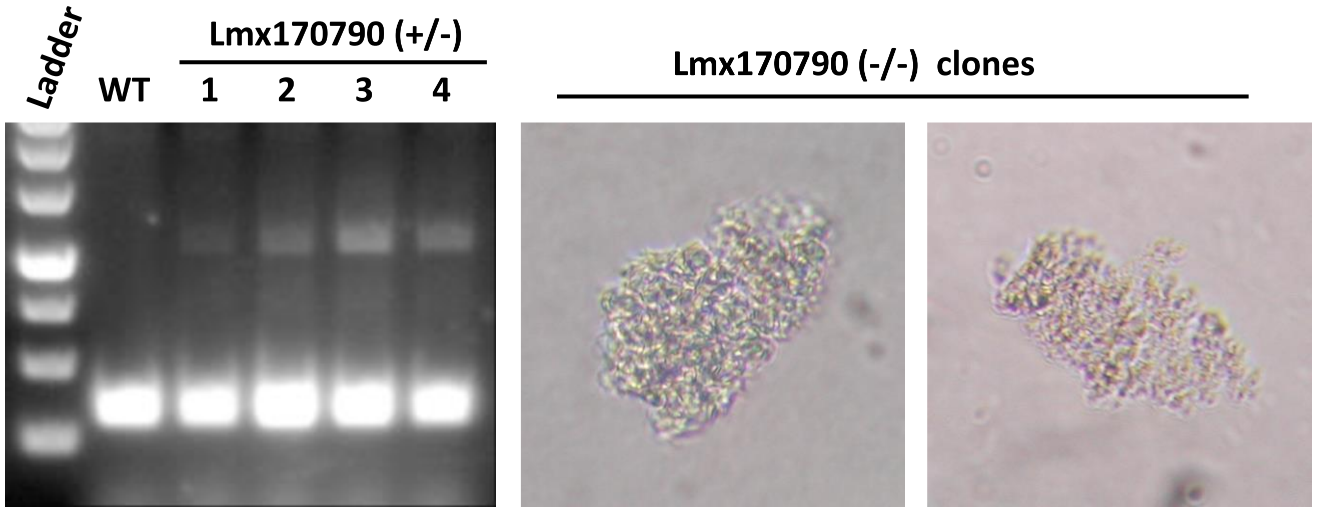
Ladder
Lmx170790 (+/-)
WT
1
2
3
4
Lmx170790 (-/-) clones

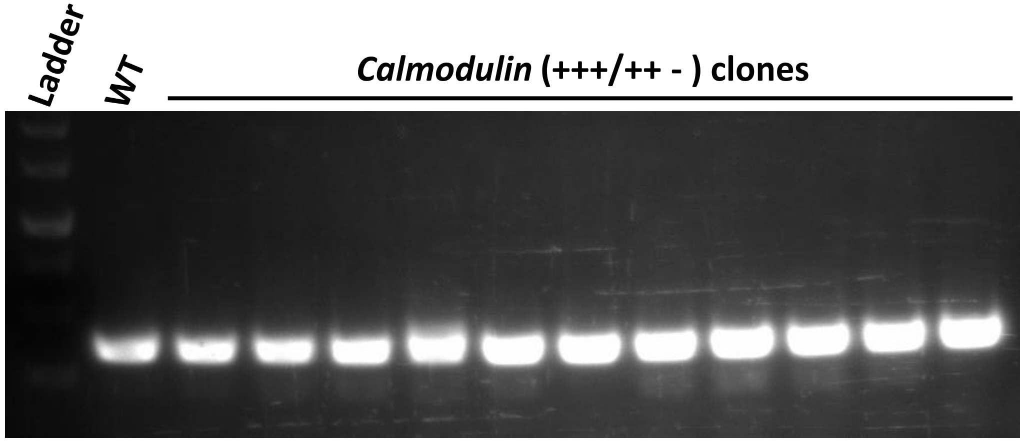

Note: likely because only one or two of the six *Calmodulin* genes was disrupted with the Bleomycin resistance gene donor in those *Calmodulin* targeting clones, the primers (L+R) would mainly amplify the 355 bp WT gene (5 copies) band, the 900 bp disrupted Calmodulin gene (1 copy) band could be barely seen in those PCR analysis

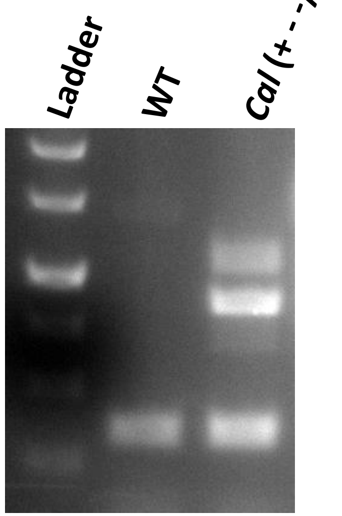

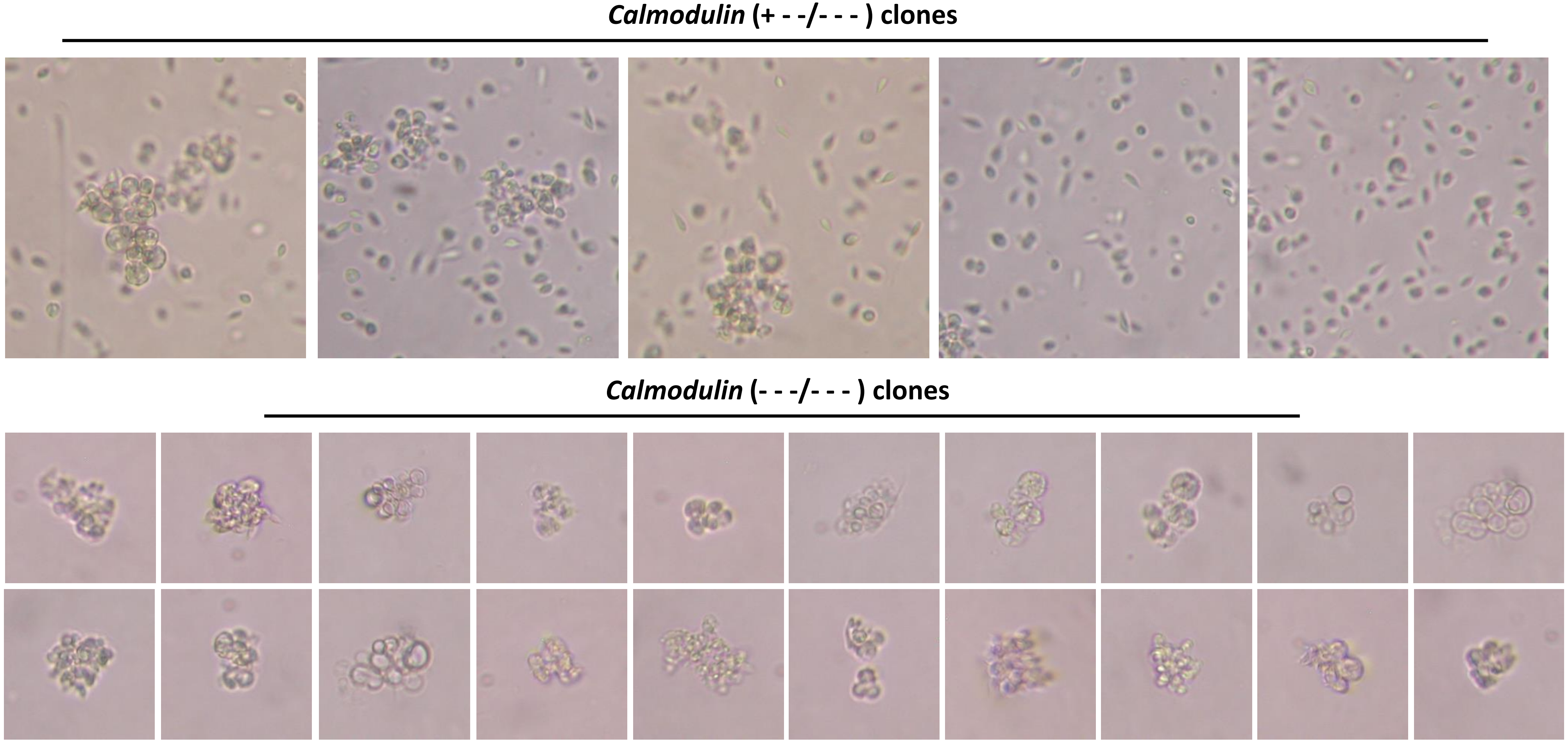

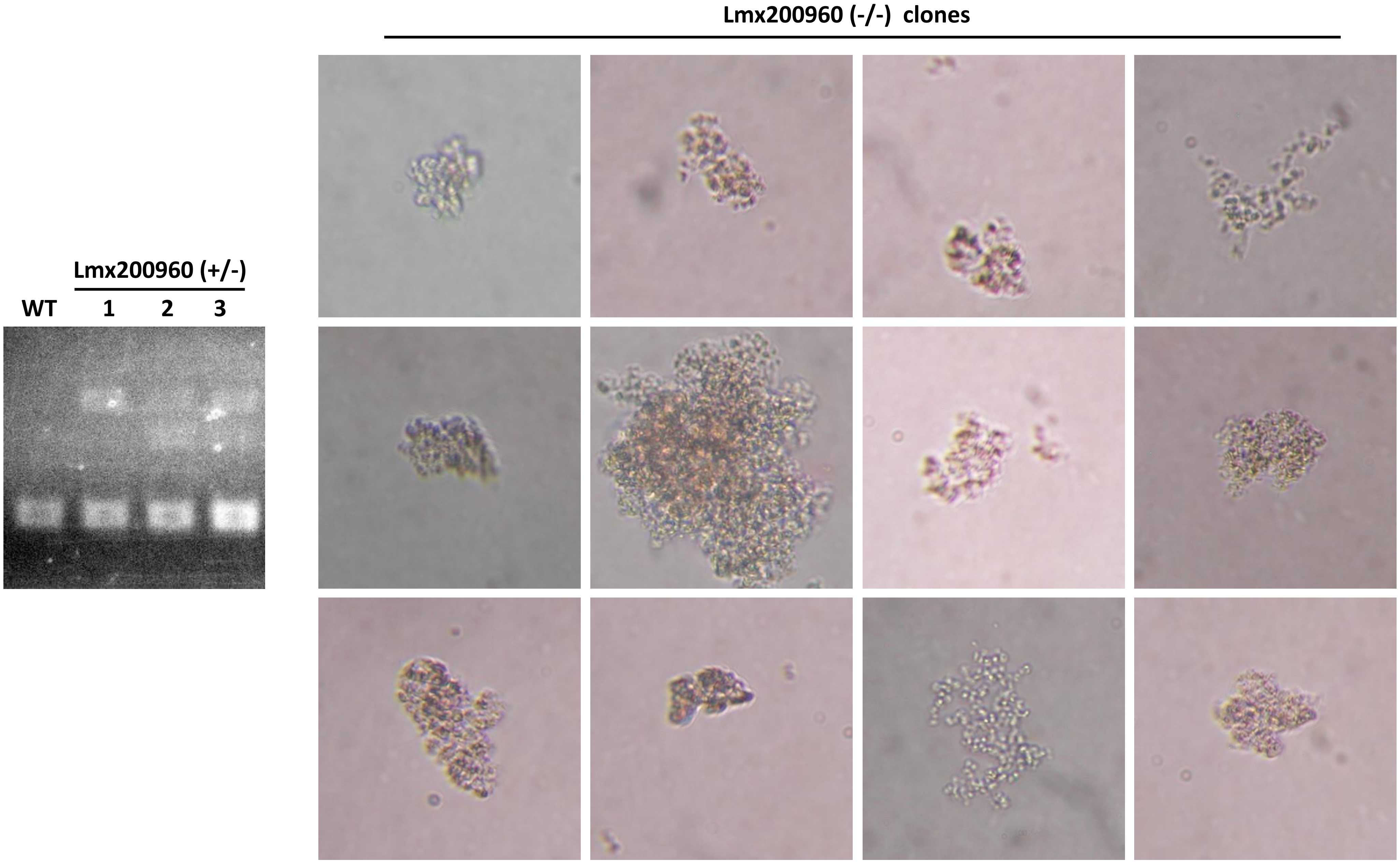
Lmx200960 (+/-)
WT
1
2
3
Lmx200960 (-/-) clones

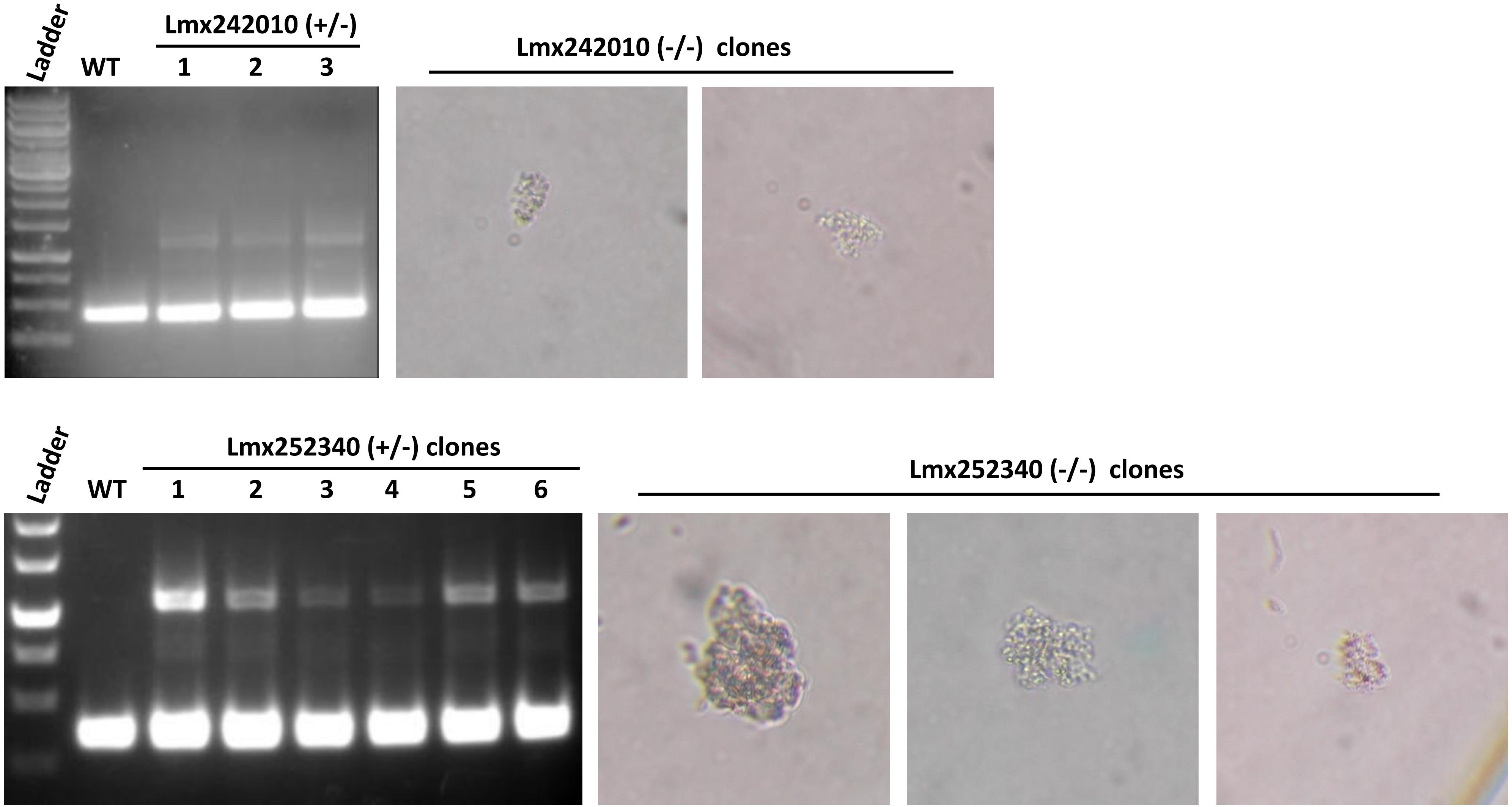
Ladder
WT
Lmx242010 (+/-)
1
2
3
Lmx242010 (-/-) clones
Ladder
WT
Lmx252340 (+/-) clones
1
2
3
4
5
6
Lmx252340 (-/-) clones

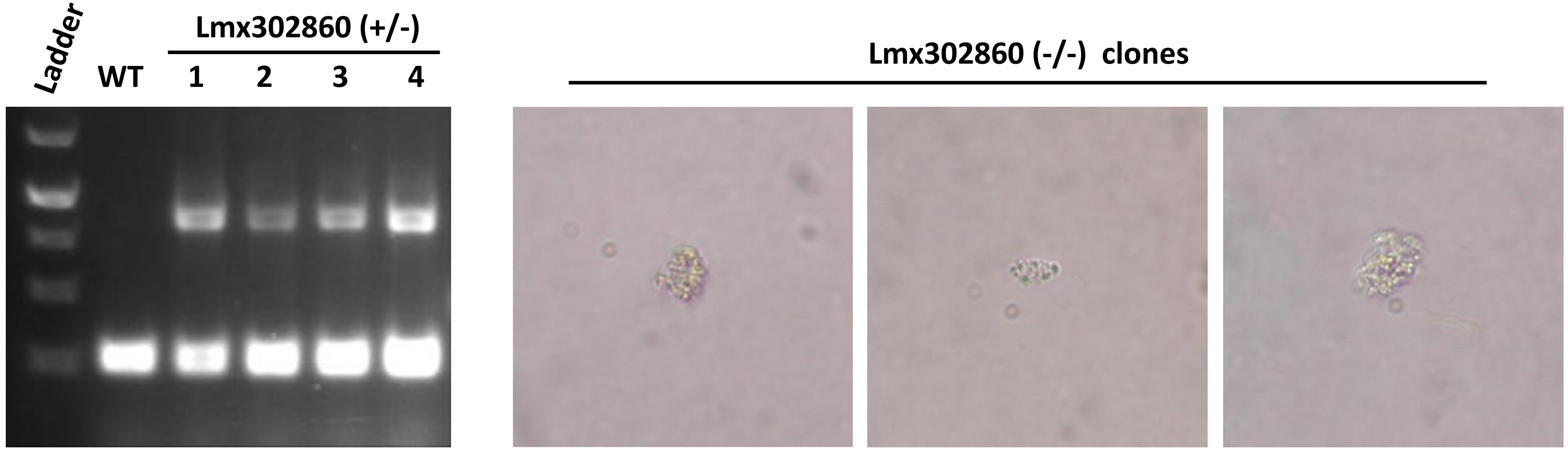

Ladder
WT
Lmx302860 (+/-)
1
2
3
4
Lmx302860 (-/-) clones

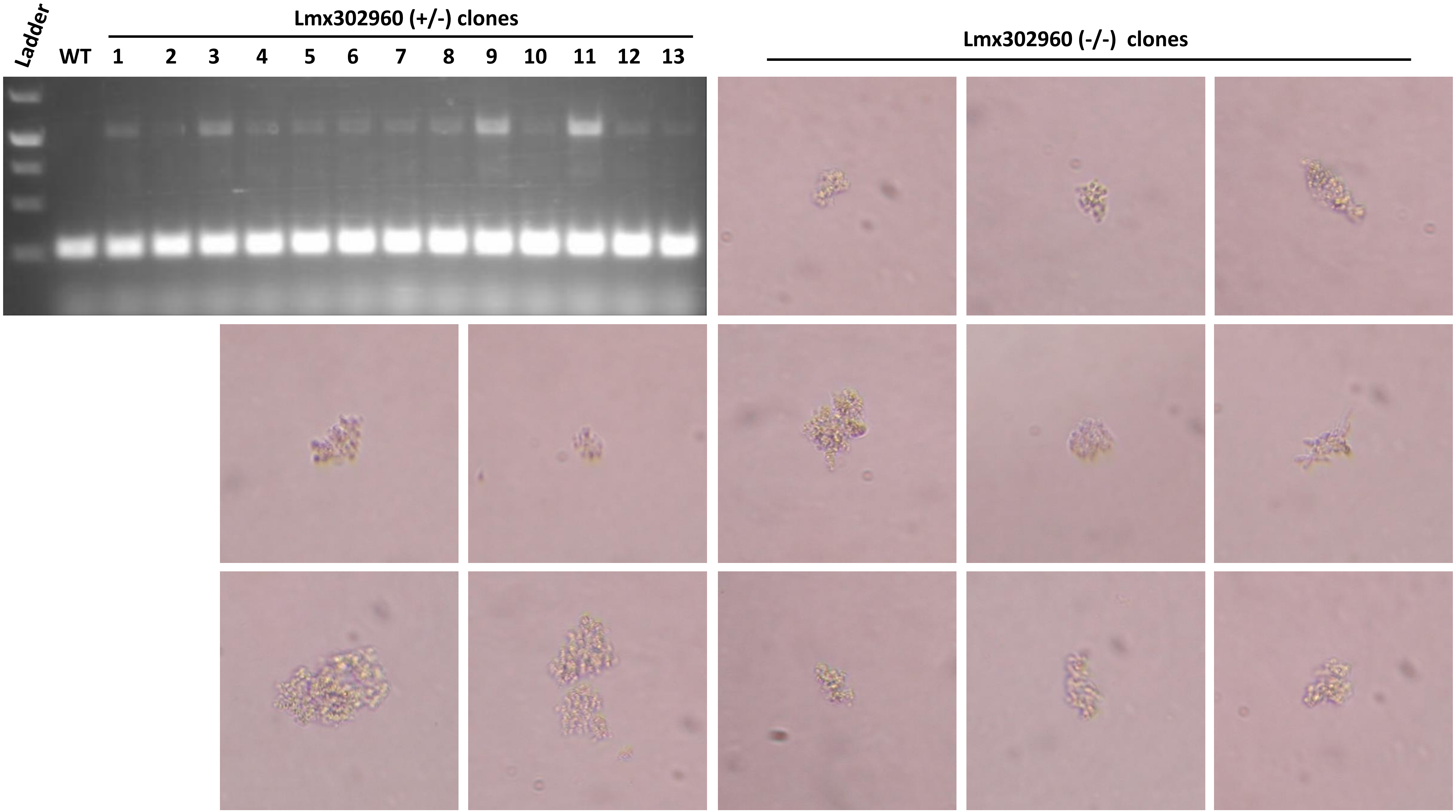
Ladder
WT
Lmx302960 (+/-) clones
1 2 3 4 5 6 7 8 9 10 11 12 13
Lmx302960 (-/-) clones

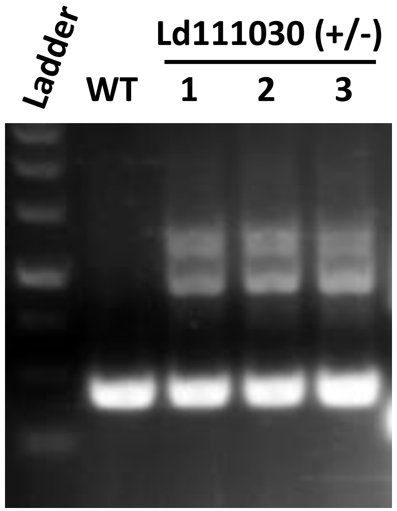
Ladder
Ld111030 (+/-)
WT
1
2
3

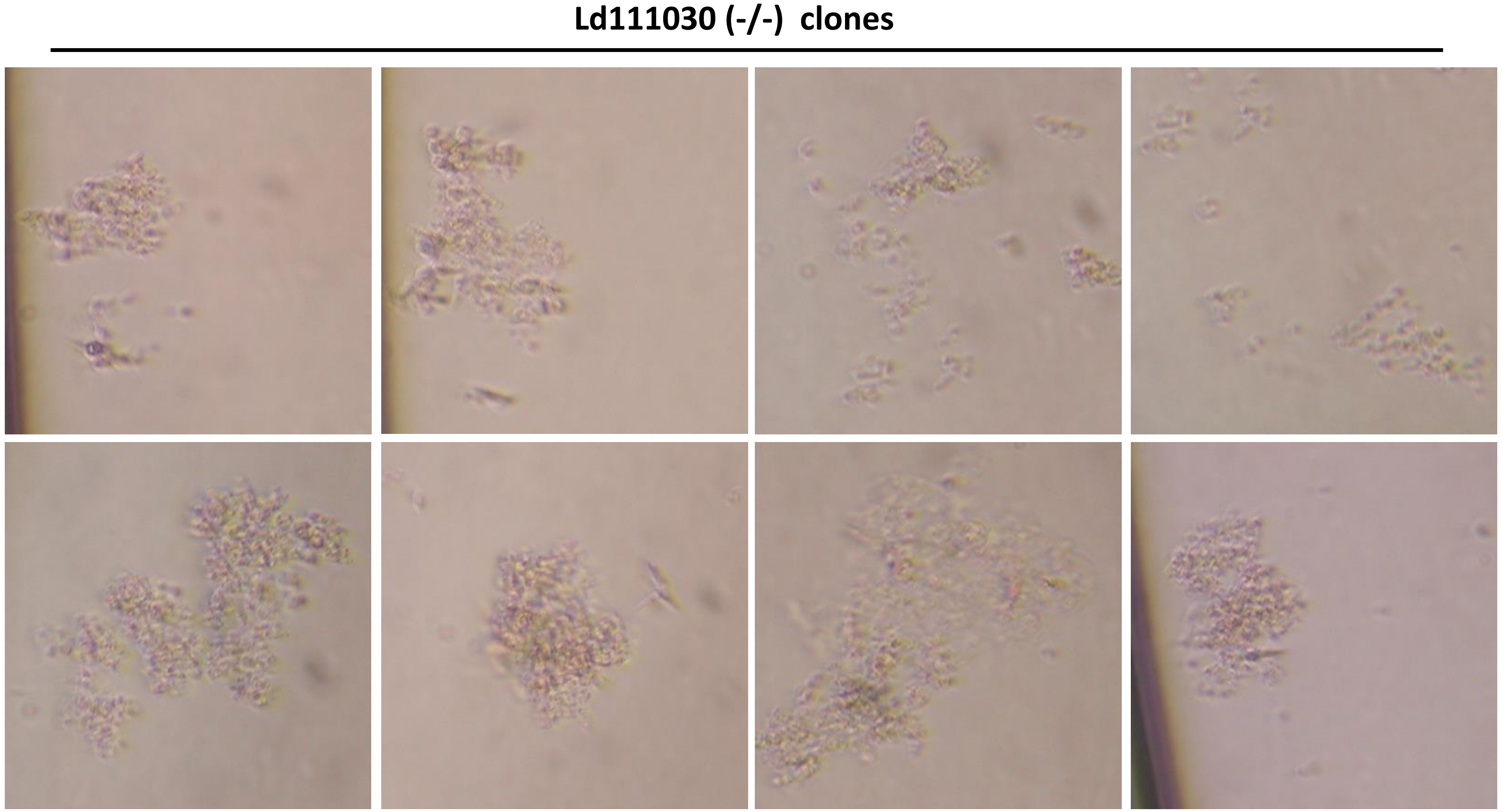
Ld111030 (-/-) clones

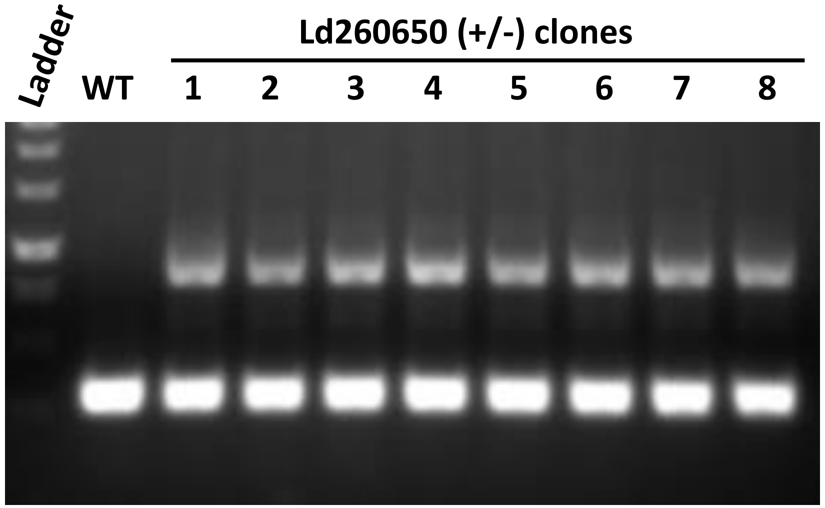

Ladder
Ld260650 (+/-) clones
WT
1
2
3
4
5
6
7
8

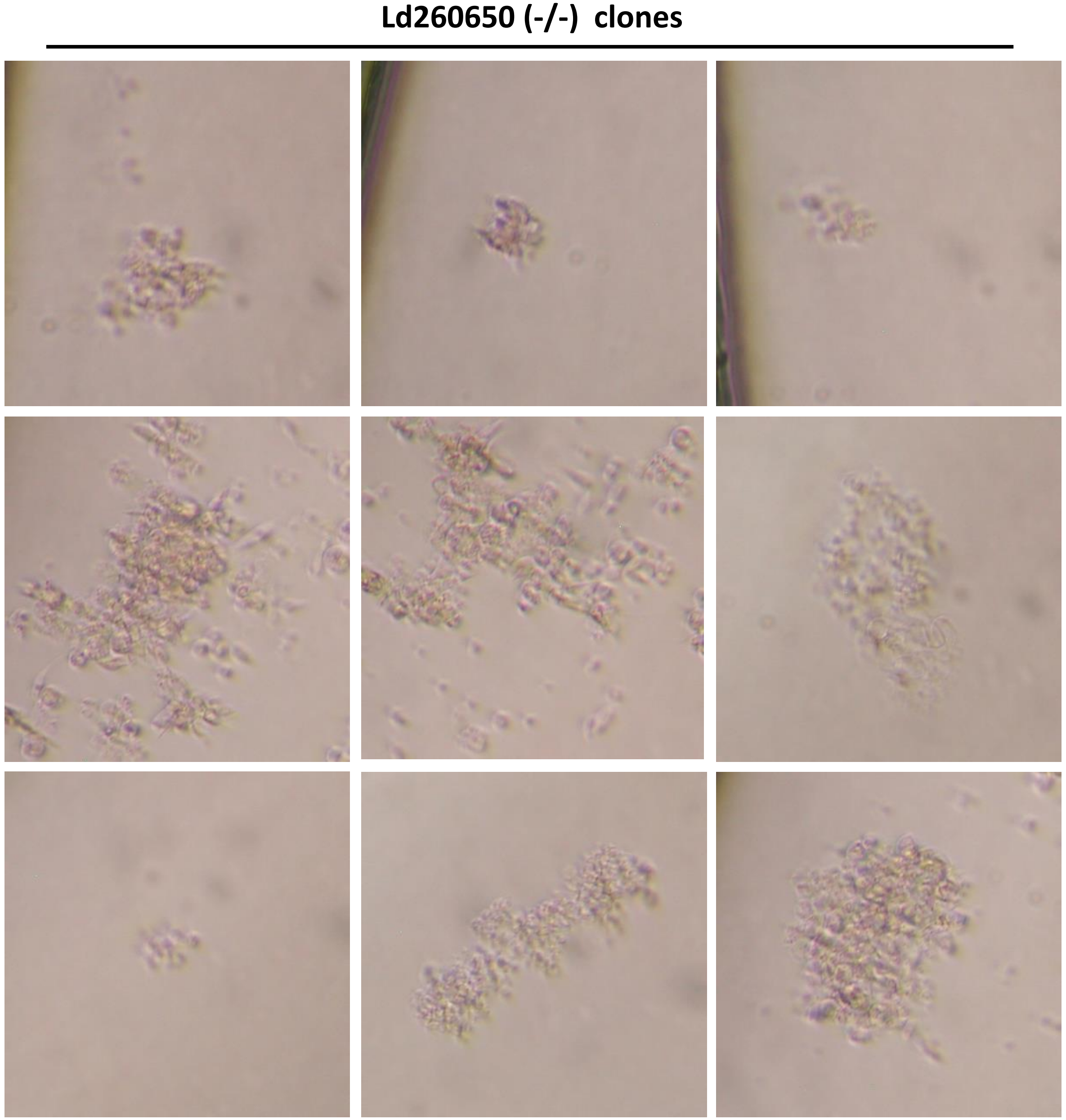

Ld260650 (-/-) clones

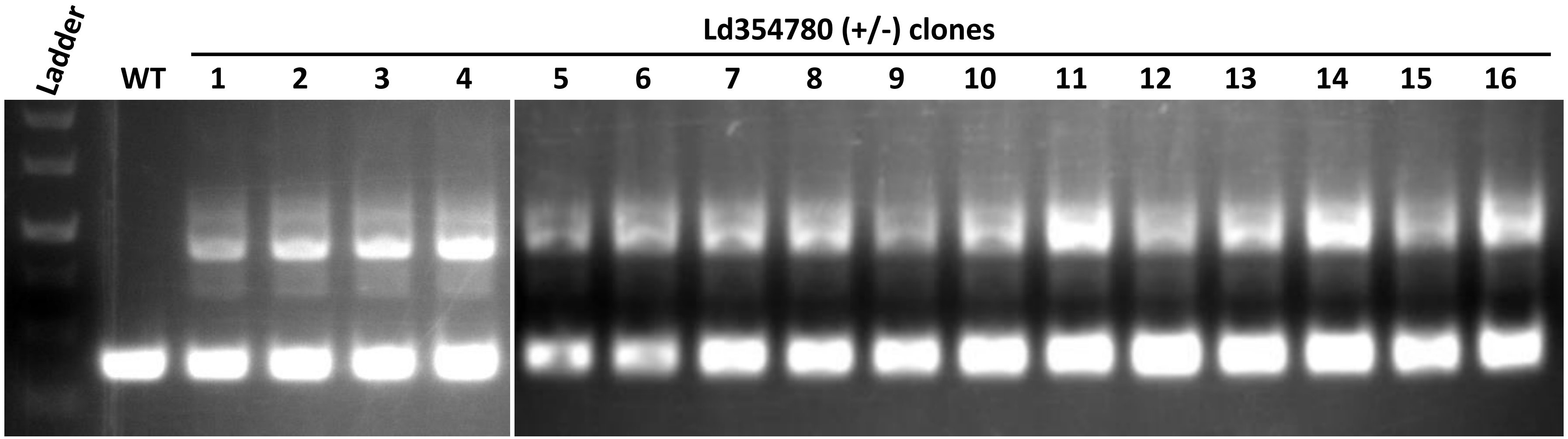

The dead cells are more spread, suggesting better mobility of the dying cells

Ld354780 (-/-) clones

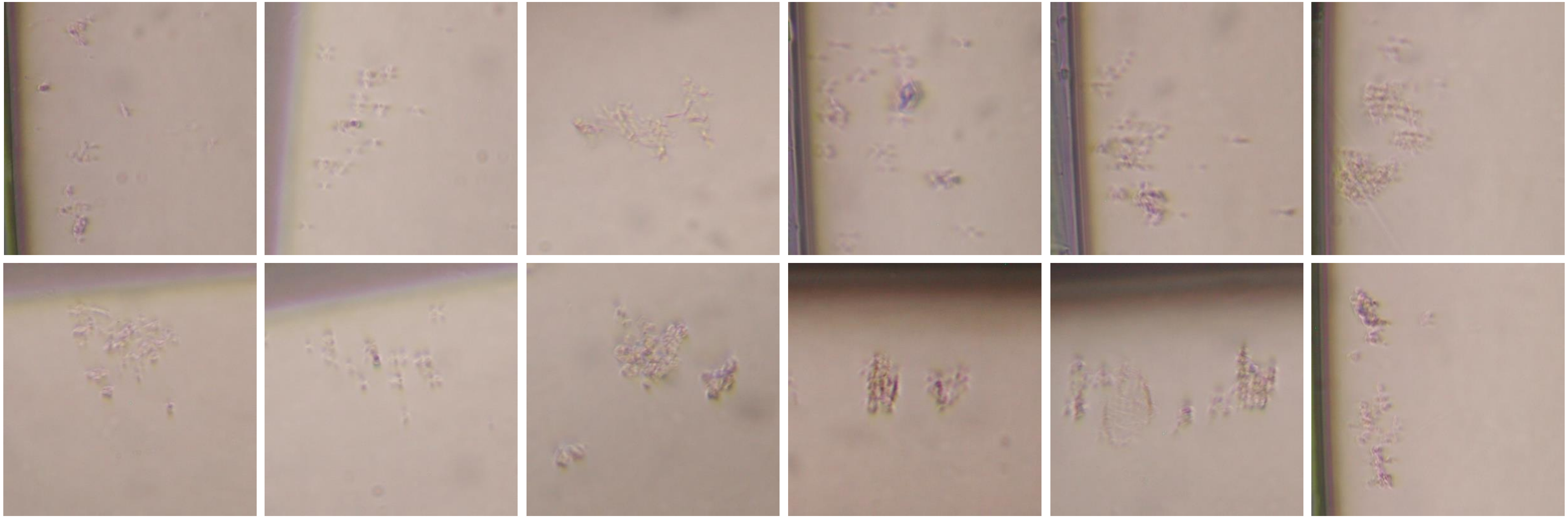

Supplement: S1 Fig — PCR shows the retention of the wild type gene for all surviving clones tested and at least one allele of the essential gene in the surviving clones (+/-) was successfully targeted and disrupted by CRISPR. Observation of the dying and dead promastigotes clumps of the essential gene null mutants’ clones (-/-) in 96 well plates. In the rRNA-P stable CRISPR protocol, the gRNA and Cas9 are constantly expressed, the gRNA/Cas9 complex will continue to scan the genome until the last copy of the target gene is deleted or disrupted. If the gene is required for viability, once the remaining copy of the essential gene has been disrupted by CRISPR after cloning into a 96 well plate, null mutants in those wells would stop growing or multiply slowly to form clumps until the gene products are diluted and degraded to the minimum level required for survival. Depending on the relative importance, and initial abundance of the essential gene product in the cell and how soon the individual promastigote was cloned into a 96 well plate after the complete gene disruption, the dying (dead) cell clump size could vary from only a few cells to more than hundreds of cells, the earlier the complete gene disruption took place before cloning, the smaller the size of the dying cell clumps was expected. In this approach, by combining observation of the dying gene null mutant clones and detection of the WT gene band in all surviving clones, using rRNA-P CRISPR protocol, we provide evidence that 14 out of 22 genes listed in Table 1 are essential for promastigote viability. Those essential genes include ten L. mexicana kinase genes: LmxM.02.0290 (Mitogen-activated kinase kinase); LmxM.03.0780 (serine/threonine-protein kinase); LmxM.08.0530; LmxM.08_29.1330 (serine/threonine-protein kinase; Aurora kinase 2, AUK2); LmxM.17.0790 (polo-like protein kinase, PLK); LmxM.20.0960; LmxM.24.2010 (phosphatidylinositol 3-kinase, PI3K); LmxM.25.2340 (AGC essential kinase 1, AEK1); LmxM.30.2860 (Tousled-like kinase, T [file pone.0316331.s001.pdf]
