# Supplementary figures and images for: Evidence for gene essentiality in Leishmania using CRISPR
Source: PLoS One. 2024 Dec 30;19(12):e0316331. doi: 10.1371/journal.pone.0316331 (PMC11684651; doi:10.1371/journal.pone.0316331)

**A**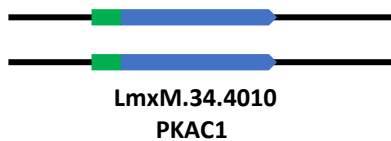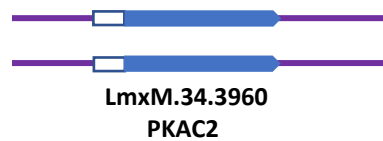**B**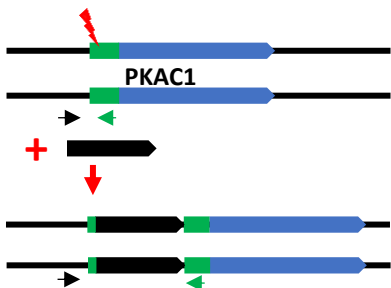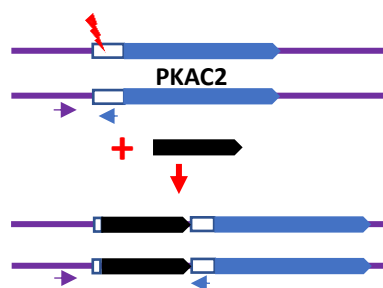**C**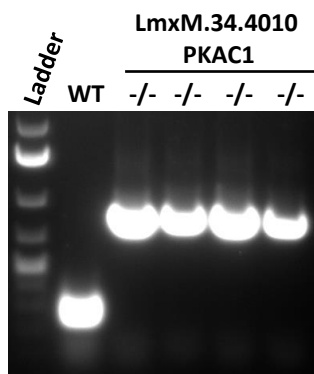**D**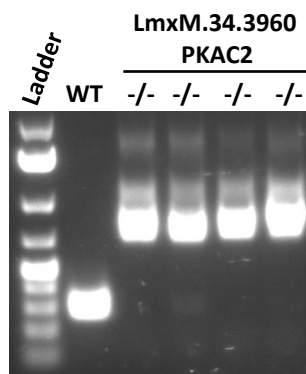

Supplement: S2 Fig — (A) PKAC1 (LmxM.34.4010) and PKAC2 (LmxM.34.3960) genes are located in chromosome 34 and share large part of conserved sequences. (B) Strategy used by rRNA-P stable protocol to disrupt LmxM.34.3960 and LmxM.34.4010 genes. Use targeting PKAC1 as an example, a gRNA was designed to target the gene specific 5’ end coding sequence of PKAC1 gene, which was then disrupted with the bleomycin resistance gene donor. (C) PCR analysis showing both PKAC1 gene alleles were successfully disrupted. (D) PCR analysis showing both PKAC2 gene alleles were successfully disrupted. (PDF) [file pone.0316331.s002.pdf]

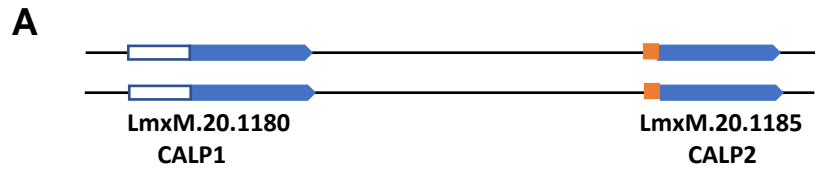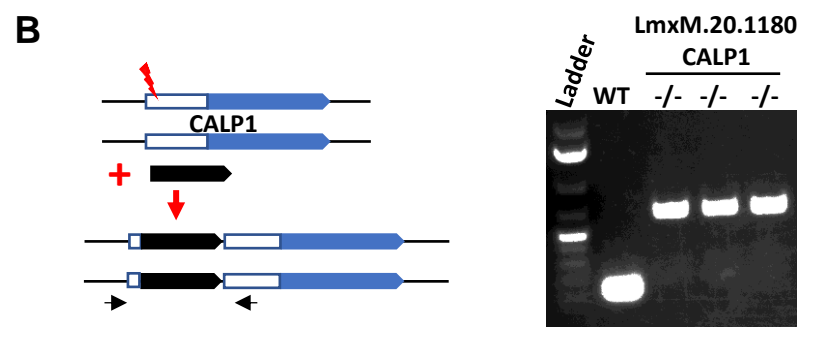

Supplement: S3 Fig — (A) CALP1 (LmxM.20.1180) gene is located in chromosome 20 and shares the conserved sequence with the downstream CALP2 (LmxM.20.11185) gene. (B) Strategy used in this study to disrupt CALP1 gene. On the left panel, a gRNA was designed to target the specific 5’ end coding sequence of CALP1 gene, which was then disrupted with the bleomycin resistance gene donor. On the right panel, PCR analysis shows both CALP1 gene alleles were successfully disrupted. (PDF) [file pone.0316331.s003.pdf]
