# Supplementary material for: Evidence for gene essentiality in Leishmania using CRISPR
Source: PLoS One. 2024 Dec 30;19(12):e0316331. doi: 10.1371/journal.pone.0316331 (PMC11684651; doi:10.1371/journal.pone.0316331)
Supplement: S1 Table — (DOCX) [file pone.0316331.s004.docx]

**S1 Table**

**Oligonucleotides and primers used in this study**

| Gene ID | Oligonucleotides and primers |
| --- | --- |
| LmxM.02.0290  (Mitogen-activated kinase kinase kinase) | Lmx020290+ 5’ TTGTACCCGATGTCGTTCATCATGA  Lmx020290- 5’ AAACTCATGATGAACGACATCGGGT  Lmx020290purF 5’CCCACCTACCCGATGTCGTTCATCACTATAGAACTCGAGCAGCTGAA  Lmx020290purR 5’TCACCGCCAGCGCAGTACTCCATCAAGACCAATGCGGAGCATA  Lmx020290L 5’ ACATGCAGACGATGCTGAAC  Lmx020290R 5’ GATGCCGTCCTCCAGTAAAA |
| LmxM.03.0780  (serine/threonine-protein kinase) | Lmx030780+ 5’ TTGTACTACACGACCGACGCCGTCG  Lmx030780- 5’ AAACCGACGGCGTCGGTCGTGTAGT  Lmx030780purF 5’TTGTTGTACTACACGACCGACGCCGCACTATAGAACTCGAGCAGCTGAA  Lmx030780purR 5’TTGCGGCTGATGGAAAACTCGTCGAAGACCAATGCGGAGCATA  Lmx030780L 5’ AGAGGAAGACACGGACAGGA  Lmx030780R 5’ GAGCACTGTCTCCGACTGCT  Lmx030780L1 5’ TTCCGCCATGGTTAGAAGTC  Lmx030780R1 5’ CGTCCTTGTCAGCGGTATCT |
| LmxM.08.0530  (protein kinase) | Lmx080530+ 5’ TTGTCGACAGCACAAAGTCCTTGTA  Lmx080530- 5’ AAACTACAAGGACTTTGTGCTGTCG  Lmx080530purF 5’ TCCGGTGCAAGTCCCGATCCGGTACACTATAGAACTCGAGCAGCTGAA  Lmx080530purR 5’ TTGTGGGCGACAGCACAAAGTCCTTAAGACCAATGCGGAGCATA  Lmx080530L 5’ AGGATTCTCCACACGTCCAC  Lmx080530R 5’ GGGTTCACACACTCACCAGTT |
| LmxM.08_29.1330  (serine/threonine-protein kinase) | Lmx8291330+ 5’ TTGTGGTGCGTGGCAAGGGTGACGA  Lmx8291330- 5’ AAACTCGTCACCCTTGCCACGCACC  Lmx8291330purF 5’ CTCGGTGAGGGGAGCTATTCGATCGCACTATAGAACTCGAGCAGCTGAA  Lmx8291330purR 5’ GACGCACGGTGCGTGGCAAGGGTGAAGACCAATGCGGAGCATA  Lmx8291330L 5’ACCCCTCCCTTGTATCTGCT  Lmx8291330R 5’TCTCAACCGACTCAGCAGTG |
| LmxM.09.0910  (Calmodulin) | Lmx090910+ 5’ TTGTGCAGCGGCACCATCGACTTCC  Lmx090910- 5’ AAACGGAAGTCGATGGTGCCGCTGC  Lmx090910BleF 5’CAGGACGGCAGCGGCACCATCGACTGATCTTCATCGGATCGGGTA  Lmx090910BleR 5’GCCATCAGCGTCAGGAACTCGGGGACGTCGGTCAGTCCTGCTCCT  Lmx090910L 5’ GCAGATCTCCGAGTTCAAGG  Lmx090910R 5’ GATCATCTCGTCCACCTCCT  Lmx090910L1 5’ GCTCATCTCCTTTGGAATGC  Lmx090910R1 5’ GCAACACACACACACAGACA  Lmx090930L1 5’ TTGGCTCTCACTCTCCTTGG  Lmx090930R1 5’ ACACACACACACACGGACAG  Lmx090930R2 5’ GGAGAAGGCGACGAGAAAGT |
| LmxM.16.1550  (Component of motile flagella 6) | Lmx161550+ 5’ TTGTAGGCGGAGTAAAGGTGAAGGC  Lmx161550- 5’ AAACGCCTTCACCTTTACTCCGCCT  Lmx161550PurF 5’ CGCTCAGGTGCCGGTCATTCGCGCCACTATAGAACTCGAGCAGCTGAA  Lmx161550PurR 5’ CCGCTGGAGGCGGAGTAAAGGTGAAGACCAATGCGGAGCATA  Lmx161550L 5’ GTGACATGGGTCTCATCACG  Lmx161550R 5’ CAGAAACACACTGCCGTCAC |
| LmxM.17.0790  (protein kinase) | Lmx170790+ 5’ TTGTGGCTCTTGTGGTCGATGATC  Lmx170790- 5’ AAACGATCATCGACCACAAGAGCC  Lmx170790purF 5’CGCCCAACTACATTGCACCCGAGATCACTATAGAACTCGAGCAGCTGAA  Lmx170790purR 5’ GTGACCGTGGCTCTTGTGGTCGATGAAGACCAATGCGGAGCATA  Lmx170790L 5’ GGACGCTGATGGACCTACTC  Lmx170790R 5’ ACGAGCAGCGTGTAGAGGAT |
| LmxM.20.0960  (protein kinase) | Lmx200960+ 5’ TTGTACTGCAGCCGCAGTCACCGAA  Lmx200960- 5’ AAACTTCGGTGACTGCGGCTGCAGT  Lmx200960purF 5’CTCTGCCACTGCAGCCGCAGTCACCACTATAGAACTCGAGCAGCTGAA  Lmx200960purR 5’CGAGAGAGTCGACACTACCCGTTTCAAGACCAATGCGGAGCATA  Lmx200960L 5’ GCAGCCAGTACGTGGAGATT  Lmx200960R 5’ TTAGAAGAGCGTTCCGTGCT  Lmx200960L1 5’ GACGATATCAGCAGCAACGA  Lmx200960R1 5’ ATTGTCGGCTGAGCAAGATT |
| LmxM.20.1180  (calpain-like cysteine peptidase) | Lmx201180c+ 5’ TTGTGGACCGAGATCGAAGAGG  Lmx201180c- 5’ AAACCCTCTTCGATCTCGGTCC  Lmx201180cBleF  5’ GAGGCCCACTGGACCGAGATCGAAGATCTTCATCGGATCGGGTA  Lmx201180cBleR  5’ ACTTCCCCTTCCTGTGGAGCCTCCTCGTCGGTCAGTCCTGCTCCT  Lmx201180cL 5’ ACCGAACCCACCATACTTGT  Lmx201180cR 5’ CGACCTCCTCTGTGAACTGT  Lmx201180cR1 5’ GGACTTCCCCTTCCTGTGG |
| LmxM.24.2010  (phosphatidylinositol 3-kinase) | Lmx242010+ 5’ TTGTATGGACAGCGTATCCAGCCA  Lmx242010- 5’ AAACTGGCTGGATACGCTGTCCAT  Lmx242010purF 5’ CGGCATGATGCCACCCATCCCATGGCACTATAGAACTCGAGCAGCTGAA  Lmx242010purR 5’ GTTGCTGTATGGACAGCGTATCCAGAAGACCAATGCGGAGCATA  Lmx242010L 5’ GCCGCCTTTCATGTCTACTC  Lmx242010R 5’ AAACGGACGCTGAGTGAAGT |
| LmxM.25.2340  (AGC essential kinase 1) | Lmx252340+ 5’ TTGTGTGCAGGCACATAATGGCCAA  Lmx252340- 5’ AAACTTGGCCATTATGTGCCTGCAC  Lmx252340purF 5’GCTGTACGGTGCCGAGATCCTCTTGCACTATAGAACTCGAGCAGCTGAA  Lmx252340purR 5’TGTGCTCGTGCAGGCACATAATGGCAAGACCAATGCGGAGCATA  Lmx252340L 5’ GCACAAGCTGTTCTTCGTCA  Lmx252340R 5’ GAAGTCCACGGAGTTGGTGT |
| LmxM.30.2860  (protein kinase) | Lmx302860+ 5’ TTGTACGCGCTGATGGCTGCCCAGA  Lmx302860- 5’ AAACTCTGGGCAGCCATCAGCGCGT  Lmx302860purF 5’TTGCAGGACGCGCTGATGGCTGCCCACTATAGAACTCGAGCAGCTGAA  Lmx302860purR 5’ GAGGCGCGCAGCTGATACTCCATCTAAGACCAATGCGGAGCATA  Lmx302860L 5’ ACGGTCAGACAGTGTGGATG  Lmx302860R 5’ AGCTTGATGTCGGCGATAGT |
| LmxM.30.2960  (Repressor of differentiation kinase 2) | Lmx302960+ 5’ TTGTGCGGTGCATCATCTTGTTCGA  Lmx302960- 5’ AAACTCGAACAAGATGATGCACCGC  Lmx302960purF 5’ CCTTGCCCTGGACCACATTCACTCGCACTATAGAACTCGAGCAGCTGAA  Lmx302960purR 5’ TCAGGTCGCGGTGCATCATCTTGTTAAGACCAATGCGGAGCATA  Lmx302960L 5’CCATCGAGAGAGAGGGACAG  Lmx302960R 5’CCTCGTACTGGCGAGAAAAG |
| LmxM.34.3960  (protein kinase A catalytic subunit isoform 2, PKAC2) | Lmx343960+ 5’ TTGTGTGCCAGCCACCTCAGGTTT  Lmx343960- 5’ AAACAAACCTGAGGTGGCTGGCAC  Lmx343960BleF2 5’ CATGCTAGTGAGTGTGGAGCCGAAAGATCTTCATCGGATCGGGTA  Lmx343960BleR2 5’ CGTGGACTGTGCCAGCCACCTCAGGCGTCGGTCAGTCCTGCTCCT  Lmx343960L2 5’ CGAACGGATACCTTTGCTCG  Lmx343960R3 5’ ACTGTGCCAGCCACCTCAGG |
| LmxM.34.4010  (protein kinase A catalytic subunit isoform 1, PKAC1) | Lmx344010+ 5’ TTGTCAAGATGTCGTCTGCTGCCA  Lmx344010- 5’ AAACTGGCAGCAGACGACATCTTG  Lmx344010BleF  5’GCAGAGCACAAGATGTCGTCTGCTGATCTTCATCGGATCGGGTA  Lmx344010BleR  5’GACTCCGCCGGATAGCTGTCCTTGGCGTCGGTCAGTCCTGCTCCT  Lmx344010L1 5’ TCCCTCCGAAGAAAGCAAGA  Lmx344010R1 5’ CTCTCAAAGGAATAGGTGGCC  Lmx344010R2 5’ CTGCTGAGTTGGCCCAATG |
| LdBPK_100590  (hypothetical protein) | Ld100590+ 5’ TTGTGAAGGCAAATCGATGGACCG  Ld100590- 5’ AAACCGGTCCATCGATTTGCCTTC  Ld100590BleF 5’ TGTGAAATGAAGGCAAATCGATGGATCTTCATCGGATCGGGTA  Ld100590BleR 5’ GCGGTGCACGTCGCCGATTCTTCGGCGTCGGTCAGTCCTGCTCCT  Ld100590L 5’ AGGCACTTCACTTTCGGTGT  Ld100590R 5’ GTATCCGATGCACCGACTTT |
| LdBPK_111030  (hypothetical protein) | Ld111030+ 5’ TTGTCTCGCATGTAATGCCGGAGG  Ld111030- 5’ AAACCCTCCGGCATTACATGCGAG  Ld111030BleF 5’ GACATACTGGACGTGTATCCGGCCTGATCTTCATCGGATCGGGTA  Ld111030BleR 5’ GTCTCTTTCTCGCATGTAATGCCGGCGTCGGTCAGTCCTGCTCCT  Ld111030L 5’GTGTCCAAGGCAACTGGTG  Ld111030R 5’TCGTCTTGGTACACGTCTCG |
| LdBPK_230540  (hypothetical protein) | Ld230540+ 5’ TTGTAAGGCTCACGGTAGACCAGC  Ld230540- 5’ AAACGCTGGTCTACCGTGAGCCTT  Ld230540BleF 5’ TCGCGGAGCTCGCGCTACTCAAGCTGATCTTCATCGGATCGGGTA  Ld230540BleR 5’ GTTGCGATAAGGCTCACGGTAGACCGTCGGTCAGTCCTGCTCCT  Ld230540L 5’GTGCCGATTTTCGCTCAT  Ld230540R 5’TGAGACTCGTGTCCATCAGG |
| LdBPK_260650  (Protein of unknown function (DUF2012)) | Ld260650+ 5’ TTGTACGGCACGTACATCTTGCAGG  Ld260650- 5’ AAACCCTGCAAGATGTACGTGCCGT  Ld260650BleF 5’ CTGCCGTACGGCACGTACATCTTGCGATCTTCATCGGATCGGGTA  Ld260650BleR 5’ AACATGAAGTAGTGGTACTCCGCCTCGTCGGTCAGTCCTGCTCCT  Ld260650L 5’TGCTCTTCGTCGTGTTATCG  Ld260650R 5’GGTGTCGCGGTACATAACCT |
| LdBPK_310120  (FG-GAP repeat protein) | Ld310120+ 5’ TTGTGATCCAGTACCTCCGTGGAG  Ld310120- 5’ AAACCTCCACGGAGGTACTGGATC  Ld310120BleF 5’ GCATGGACGACCTGACACTCACCTCGATCTTCATCGGATCGGGTA  Ld310120BleR 5’ ACGCCGCTGATCCAGTACCTCCGTGCGTCGGTCAGTCCTGCTCCT  Ld310120L 5’ ACTGGGTGAAGGACAACCTG  Ld310120R 5’ GACTGAGGTGGCTGTTTGGT |
| LdBPK_312380  (3'-nucleotidase/ nuclease) | Ld312380+ 5’ TTGTACACGGACGAAGACTTCACC  Ld312380- 5’ AAACGGTGAAGTCTTCGTCCGTGT  Ld312380BleF 5’ GCCGTACTACACGGACGAAGACTTCGATCTTCATCGGATCGGGTA  Ld312380BleR 5’ TCTGTACGGGGCTGACATCCAAGGTCGTCGGTCAGTCCTGCTCCT  Ld312380L 5’AGGTGCTGTCCTTCTCTGGA  Ld312380R 5’CTGGACGATAACGTCGGAGT |
| LdBPK_354780  (Hsp70 protein) | Ld354780+ 5’ TTGTACTGCAAAACCACTGCTGTG  Ld354780- 5’ AAACCACAGCAGTGGTTTTGCAGT  Ld354780BleF 5’ GCTGTCGTACTGCAAAACCACTGCTGATCTTCATCGGATCGGGTA  Ld354780BleR 5’ TTGGATCCACAACATCATCCTTCACGTCGGTCAGTCCTGCTCCT  Ld354780L 5’ TTCCCTTTTTCCGTGATTTG  Ld354780R 5’ TCAGTATGCGTTGAGCCAAG |
