# Supplementary material for: Evidence for gene essentiality in Leishmania using CRISPR
Source: PLoS One. 2024 Dec 30;19(12):e0316331. doi: 10.1371/journal.pone.0316331 (PMC11684651; doi:10.1371/journal.pone.0316331)

Fig 1B

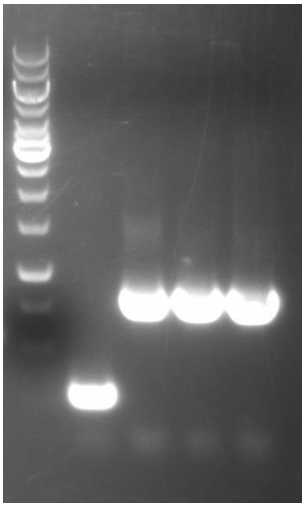

Fig 4A and Fig 1C

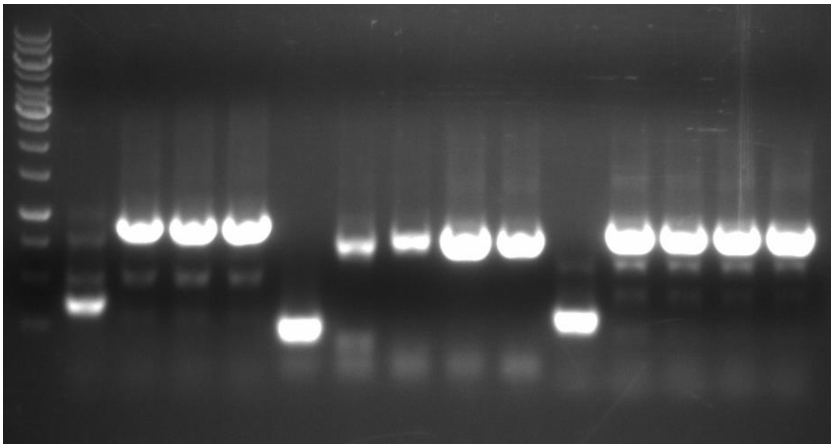

Fig 2B

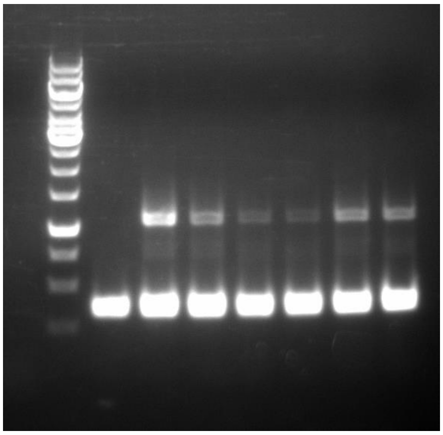

Fig 4B

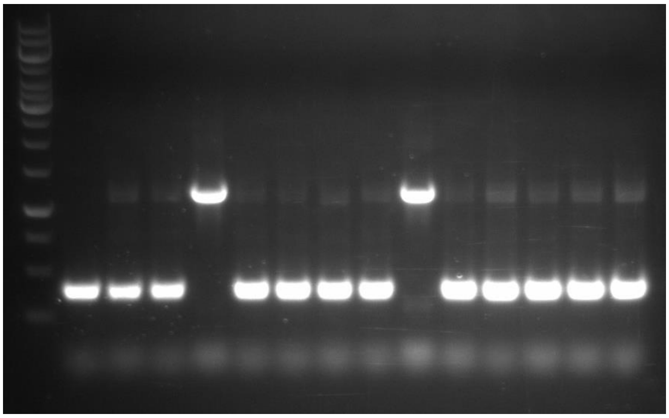

Fig 3C

Lmx090910R1+30L1

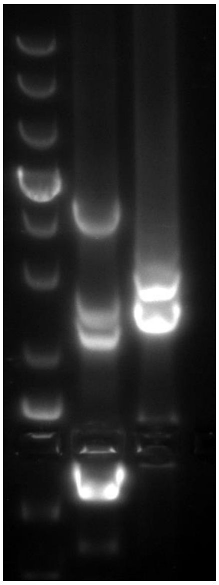

Lmx090910LR

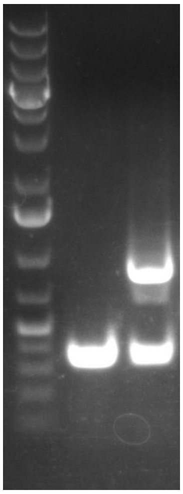

Lmx090910L1+R

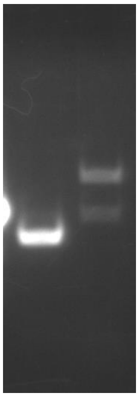

Lmx090930R1+L

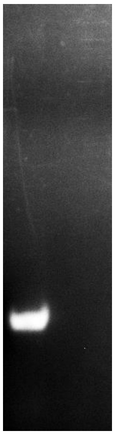

Lmx090930L1+R

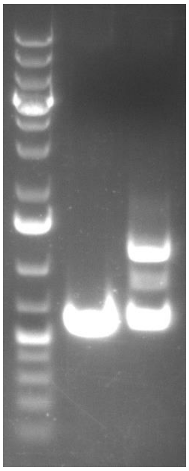

S1 Fig

Lmx020290 (+/-) clones

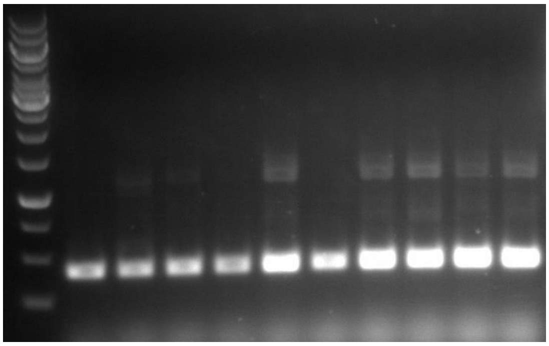

Lmx030780 (+/-) clones

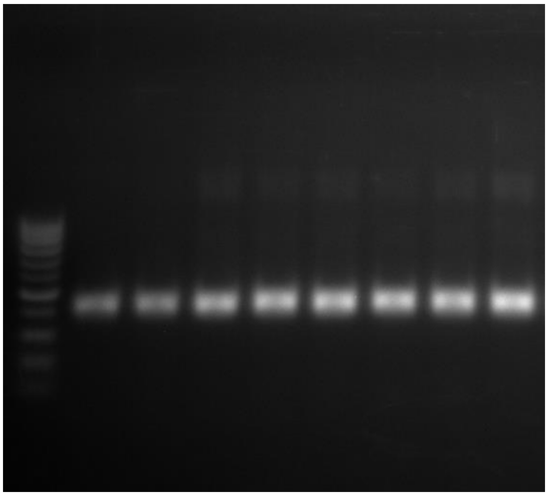

Lmx080530 (+/-) clones

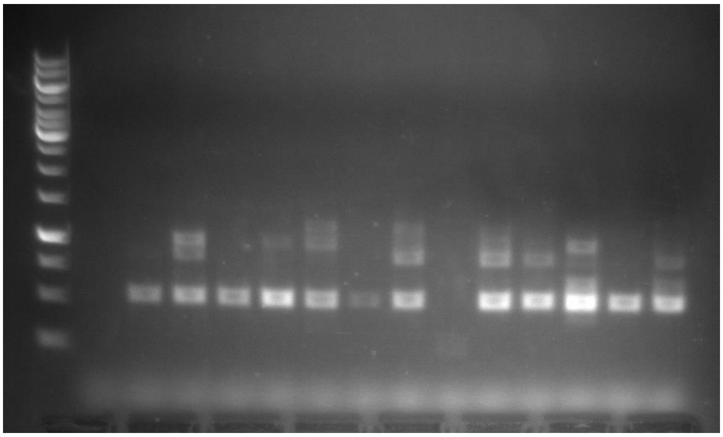

Lmx8291330 (+/-) clones

x x x x x x wt 1 2 3 4

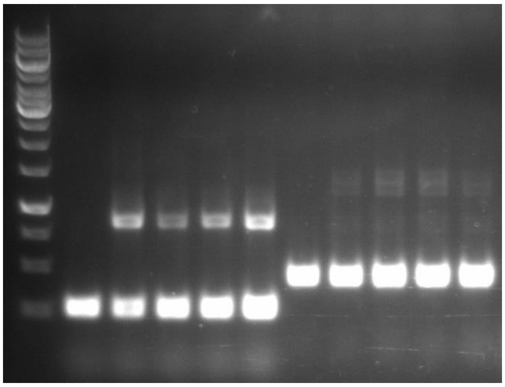

Lmx170790 (+/-) clones

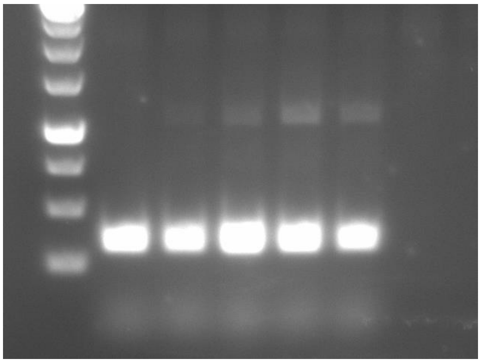

***Calmodulin* (+--/---) clone**

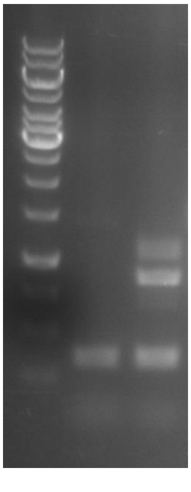

***Calmodulin* (+++/++-) clones**

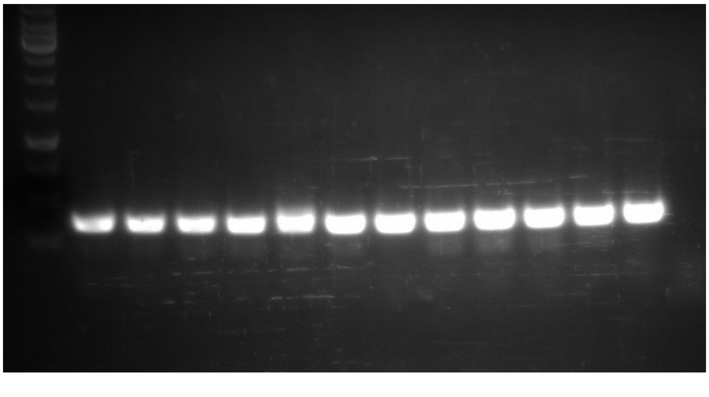

Lmx200960 (+/-) clones

X X WT 1 2 3

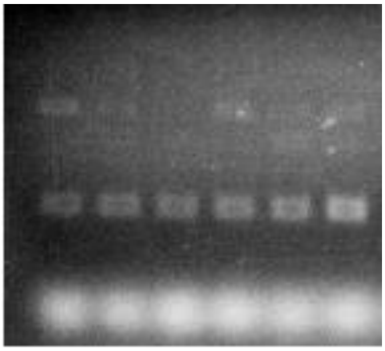

Lmx242010 (+/-) clones

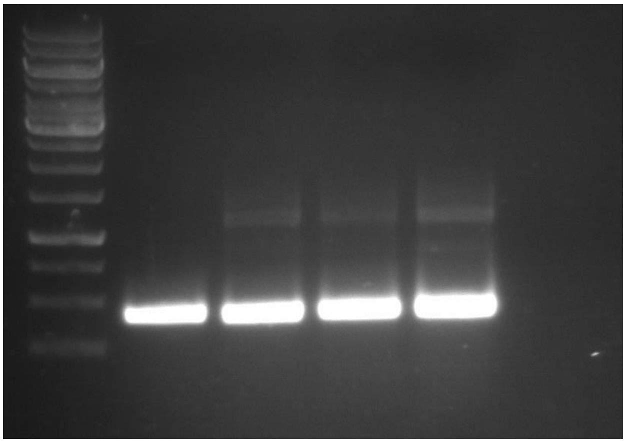

Lmx252340 (+/-) clones

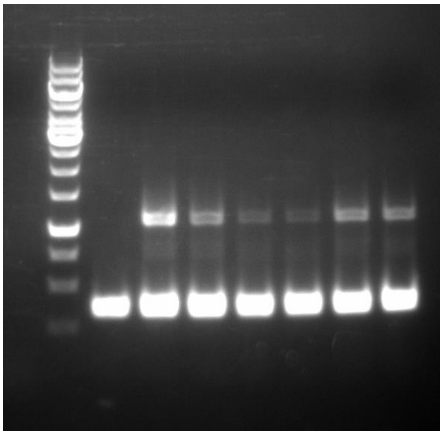

Lmx302860 (+/-) clones

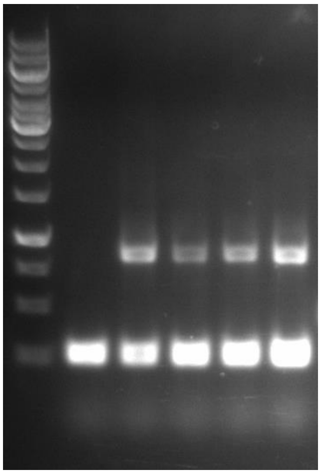

Lmx302960 (+/-) clones

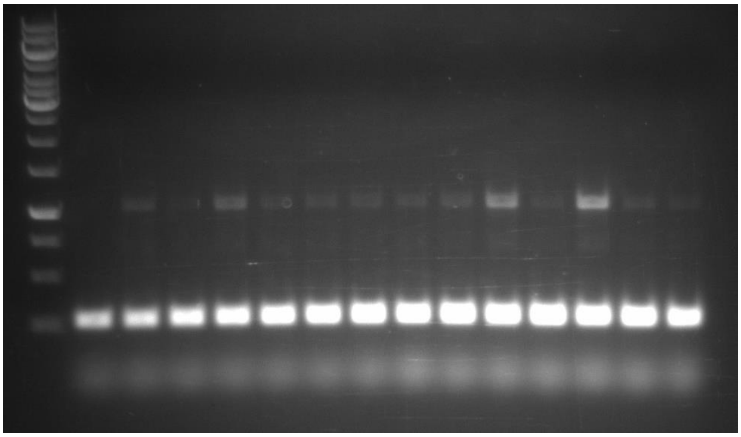

Ld111030 (+/-) clones

WT 1 2 3 x x x x x x x

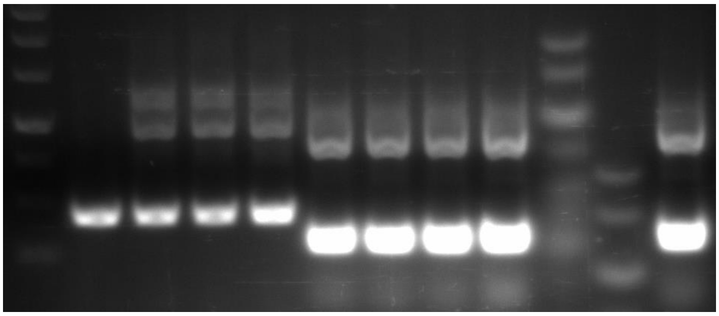

Ld260650(+/-) clones

x x x x x WT 1 2 3 4 5 6 7 8

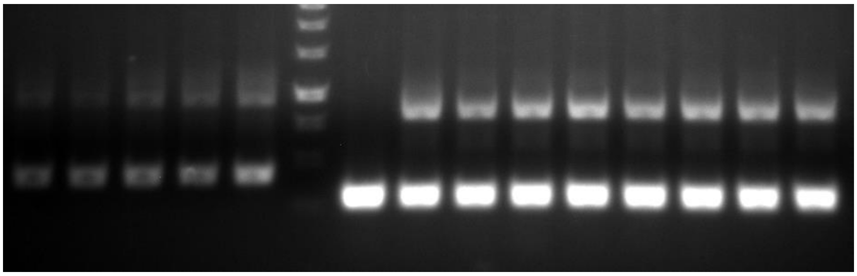

Ld354780 (+/-) clones

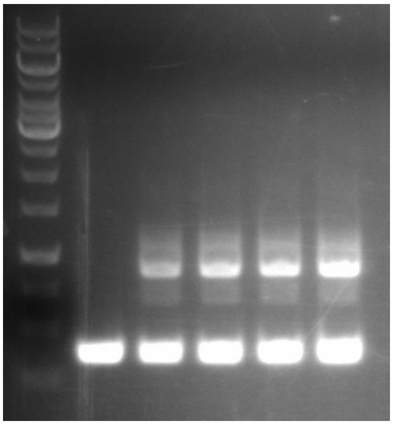

Ld354780 (+/-) clones

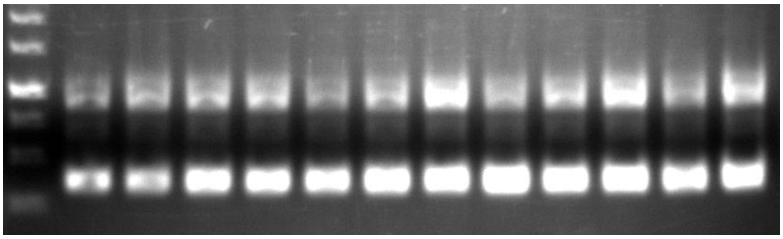

S2C Fig

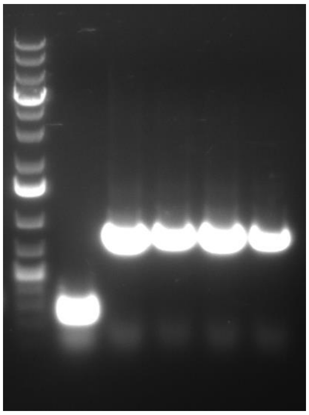

S2D Fig

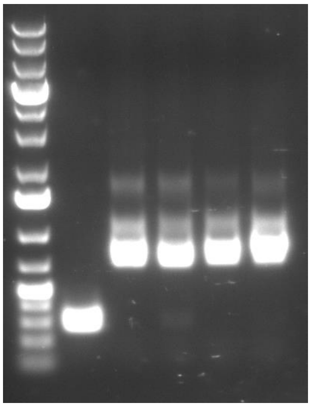

S3B Fig

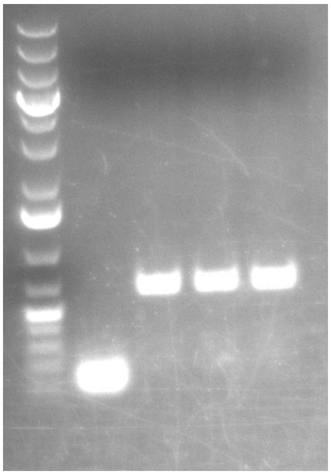

Supplement: S1 Raw images — (PDF) [file pone.0316331.s010.pdf]
