# Supplementary material for: Evidence for gene essentiality in Leishmania using CRISPR
Source: PLoS One. 2024 Dec 30;19(12):e0316331. doi: 10.1371/journal.pone.0316331 (PMC11684651; doi:10.1371/journal.pone.0316331)
Supplement: Attachment — Submitted filename: Response to reviewers.docx [file pone.0316331.s011.docx]

**Responses to Editor Dr. Kelly**

**Editor Comment.** At the heart of this work is a comparison of null-mutant generation between two different CRISPR methodologies, the transient T7 approach and the stable rRNA promoter-driven gRNA approach. It is interesting and somewhat informative that in several instances, the stable approach has successfully yielded null mutants, where the transient approach targeting the same gene was unsuccessful. However, this work would have been stronger if the authors performed a direct, side-by-side comparison of the two techniques instead of comparing their stable approach data to the published outcomes of transient-based methodologies. This point is discussed in detail by Reviewer 2.

**Response**: We agree it is better to perform a direct, side-by-side comparison between the T7 transient protocol and the rRNA-P stable protocol. Ideally, the direct side by side comparison should be carried out by a third-party lab to avoid bias since our lab developed the rRNA-P stable protocol. Nevertheless, the T7 transient protocol studies (20-22) were performed from well-established *Leishmania* research labs with a minimum three or more attempts for each of the genes outlined in Table 1. Consequently, we do not believe the outcome would be different if these analyses were repeated in our lab. See also further explanations in responses to Reviewer 2 below.

**Editor Comment**: In line with concerns raised by Reviewer 2, the authors conclude that “dying and dead cells were caused by the disruption of all wild type gene allele present in these clones”, however presumably due to the obvious difficulties inherent with this, they do not provide direct supportive evidence.

**Response**: We understand this concern. We have attempted to extract the genomic DNA from those dying and dead Leishmania cells in the wells of 96 well plates. However, due to the extreme low yield and poor quality (degraded DNA), we were not able to obtain PCR products. We reasonably believe the disruption of all wild type essential gene alleles in these clones is the most likely cause of the cell dying and death. To better explain and illustrate how gene essentiality is normally determined with the rRNA-P stable protocol, we have now rewritten the section of essential genes (line 170-232) using the AGC essential kinase 1 gene (LmxM.25.2340, AEK1) as a typical example (see new Fig. 2).

See also response below to reviewer 2 that further explains the interpretation of dead and dying clones.

Different from the T7 transient protocol, the gRNA and Cas9 are constantly expressed in the stable CRISPR protocol. Once the CRISPR plasmid pLdCN (pLdsaCN) was transfected into *Leishmania* promastigotes, the gRNA/Cas9 complex would continue scanning the genome until all the WT alleles were targeted and disrupted. If the gene is required for viability and once the remaining copy of the essential gene has been disrupted by CRISPR, cells in those wells would stop growing or multiply slowly to form clumps until the gene products (mRNA and protein) are diluted and degraded to the minimum level required for survival. Depending on the relative importance, initial abundance, and stability of the gene product in the cell and the cloning time (stage) post the complete gene disruption for the individual clone, the dying (dead) cell clumps could contain only a few cells to more than hundreds of cells (new Fig. 2A and see below). (Note: if the cell dying was caused by the failed double strand DNA break repair, the promastigote would stop proliferating right after cloning and would not be able to form clumps containing more than ten to hundreds of cells; if the cell dying was caused by a spontaneous rare lethal mutation in the genome (unlikely), much fewer dying clones (maybe only one clone if it did happen) would be observed in a 96 well plate, instead of more than three dying clones regularly observed for disruption of an essential gene). PCR analysis will show the WT gene band persists in all surviving clones and at least one allele of the essential gene was successfully targeted and disrupted by CRISPR (new Fig. 2B). In this manner by combining observation of death of gene null mutant clones and detection of the WT gene band and the gene targeting band in all surviving clones, using rRNA-P stable protocol, we were able to confirm with confidence that many of those genes which T7 system was not able to generate alive null mutants (14 out of 22, see table 1, new Fig 2, new Fig 3 and S1 Fig ) are truly essential for *Leishmania* viability.

**Editor Comment:** The LmxM.20.1180 null mutant was described in the text as normal in mobility, yet the swimming assay data in Fig 5B indicate that it is more than twice as mobile as WT. This should be accounted for.

**Response**: We agree that a more precise description should be added to the text regarding LmxM.20.1180 null mutant. We have now revised the sentence (line 297-299) as below:

In comparison, the LmxM.20.1180 (CALP1.1) null mutants are normal in size but appeared to be able to proliferate and swim slightly faster than the wild type cells (S5 movie and Figs 5 and 6).

**Editor Comment:** In Fig. 2D, please account for the additional fainter band running at 700 bp in the Cal (+--/---) lane.

**Response**: For simplicity we did not include the detailed PCR analysis data in the old Figure 2 in the version submitted. We have now rewritten the text regarding the *Calmodulin* gene targeting and included the detailed PCR analysis data on the *Calmodulin* (+--/---) clone with additional PCR primers in the new Figure (now Figure 3) (line197-232). As you can see in Fig. 3, PCR analysis revealed that CRISPR gene targeting had deleted both alleles of LmxM 09.0930, disrupted both alleles of LmxM 09.0910 and one allele of LmxM 09.0920, and only one wild type LmxM 09.0920 allele remained in this slowest growing clone (Fig. 3B&C). It is interesting to note: a smaller than the expected size (R+10R1) band was detected in this Cal (+--/---) clone, indicating a recombination deletion had occurred in one of the two disrupted LmxM 09.0910 alleles, which also explains the additional fainter band running at 700 bp detected in the PCR with (R+L) primer pair.

**Editor Comment:** Many minor issues with language throughout the manuscript that need to be edited.

**Response**: We have carefully gone through the manuscript and made corrections if necessary.

**Editor Comment:** In addition to these points, please also address all points raised by Reviewer 2.

**Response**: We have responded to all concerns raised by Reviewer 2.

**Reviewer 1**

**Comment:** This is a timely and very important paper that provides important information on one of the most used techniques: gene deletion. Moreover, we, the scientists in the field of leishmaniasis, struggle to be sure about the essentiality of the gene we delete from the parasite genome. To date, CHRISPR is the most used technology for gene deletion. This study provides an important and useful guide on this topic. The paper is well written and describes a carefully designed study. Hence, this paper should be accepted for publication as is.

**Response**: We thank Reviewer 1 for his understanding and support of this study.

**Reviewer 2**

**Comment**: This paper reports the results of gene deletion attempts with a specific CRISPR protocol (named rRNA-P “stable” protocol). 22 genes were targeted, which in previous studies that used a different CRISPR protocol (“transient” protocol) did not yield confirmed null mutants. The authors show that they successfully knocked out some of these genes, and for some they also failed to obtain null mutants.

**Response**: We agree.

**Comment:** The stated aim, broadly, is to determine whether the “stable” protocol could be used to “define genes as essential in Leishmania”. The authors conclude that “This study demonstrates the advantage of performing the rRNA stable protocol to confirm gene essentiality […].” (line 128) This conclusion is flawed. Fundamentally, failure to generate a viable knock-out is never sufficient to prove that said gene is “essential”. Jones et al. 2018 (PMID: 29384366) have provided the most comprehensive analysis to date of reverse genetically engineered knockout mutants in Leishmania. They rehearsed in detail how a combination of different genetic approaches can increase the confidence with which a claim of “essentiality” can be made. That landmark study was not cited, but these considerations are highly relevant.

**Response**: The literature review article by Jones et al. 2018 (PMID: 29384366) proposes a 5-star method for determining gene essentiality which we generally agree with and have now included this reference in the revised paper (ref 2). In practice however, it is not practical to meet the 4–5-star stringent criteria proposed by Jones et al using forced plasmid shuffling and DiCre gene deletion methods which are labor intensive and time consuming, for which there are only a few examples in the literature for *Leishmania.* Actually, the rRNA-P stable CRISPR is similar to the DiCre inducible gene deletion method as both methods rely on observation of cell death after complete disruption or deletion of the essential genes.

Because of its simplicity and effectiveness, CRISPR has revolutionized gene targeting methods in almost all living organisms. We believe it is also time to embrace the new CRISPR technology for determining gene essentiality in Leishmania. For example, Dr. J.C. Mottram group, the authors of the above review article, has set up a new diagnosis standard for essential genes with the T7 CRISPR transient protocol; that is a gene can be considered essential if the alive null mutant could not be generated after minimum three attempts of the T7 transient method (see nature communication (reference 21) “If a gene deletion mutant was unable to be identified after three transfection attempts, it was classed as ‘required’ with a 1-star quality classification. Forty-three protein kinases and AMPKγ fell into this category”) [21]. As verified by our stable CRISPR protocol in this study, three attempts’ method did correctly predict the gene essentiality for 10 of the 12 kinase genes which the T7 transient protocol failed to isolate the alive null mutants after three attempts in reference 21 study. If the PKAC1 and PKAC2 are excluded because failure to generate those null mutants were likely due to the primer design error in the references 20 and 21 studies, the predication accuracy is 100% (10/10). However, verified by this study, this three attempts’ method has only 43% (3/7) accurate rate in predicting the gene essentiality for 7 L. donovani genes from reference 22 study. Therefore, we believe it is important to understand the advantages and weaknesses of the T7 transient method and the rRNA-P stable method in targeting Leishmania genes and determining their essentiality. As demonstrated in this study, (the exact statement in line 128) “These results suggest that the rRNA-P stable protocol is a useful complement to the T7 transient system for investigating gene essentiality and may be more suitable for targeting multicopy genes.”

This mentioned reference (below) has now been cited as reference 2

Jones, N. G., Catta-Preta, C. M. C., Lima, A. P. C. A. & Mottram, J. C. Genetically validated drug targets in *Leishmania*: current knowledge and future prospects. *ACS Infect. Dis.* **4**, 467–477 (2018).

Consistent with the Jones review article (ref 2) we have changed the title of the paper to: “Evidence for gene essentiality in *Leishmania* using CRISPR”

Regardless of how essential genes are classified in *Leishmania,* this paper highlights one important consideration. As described, the rRNA stable protocol should be considered to generate the null mutant when it is not possible to generate a null mutant with the T7 transient protocol. The ability to generate null mutants is essential for studying differentiation, virulence, pathogenesis and basic biology of the parasite. This is now highlighted in the discussion of the revised version on lines 338-342.

**Comment:** The attempts to compare the “stable” and the “transient” methods are flawed too. Using the two methods in parallel to target the same genes and compare results would allow for a comparison. Instead, results were taken from studies with different goals (generating large numbers of KOs, prioritising throughput) and compared here against the goal of isolating null mutants for a small handful of genes. This is a comparison of two very different experimental workflows from which few conclusions can be drawn about the relative power of these methods to achieve gene knockouts. The key difference is clonal vs. population analysis, rather than “stable” vs. “transient” CRISPR methods.

**Response:** As our response to editor, to avoid preference, ideally, comparison between the stable and transient CRISPR methods should be performed in parallel to target the same batch of genes by a third-party laboratory.

We understand generating hundreds of Leishmania gene Knockouts with the T7 transient method were challenging in the previous three studies (see reference 20,21and 22). However, it is not true that less effort was put to isolate the individual gene null mutants in those three high throughput studies. As mentioned above, a minimum of three transfection attempts of T7 transient protocol were performed in order to class a gene as essential according to the three attempts diagnosis standard (see reference 21). In fact, 5 transfections were attempted for the non-essential L. donovani genes LdBPK_100590 and LdBPK_230540 but the T7 transient method somehow failed to generate the null mutants. Furthermore, for unknown reasons, the T7 transient method was still not able to generate the chromosome alleles deletion mutant for LdBPK_100590 gene even after an episomal copy of the gene was provided to the parasite (see Fig. 2 in reference 22).

For LmxM.34.4010 gene (PKAC1), likely because both the authors of reference 20 and 21 (two of the best Leishmania research labs in the world) did not realize that PKAC1 and PKAC2 (LmxM.34.3960) possess a large identical sequence at 3’ end (see S2 Fig), the primers used to verify the gene deletion in reference 20 and 21 were from the sequence shared by both PKAC1 and PKAC2. Therefore, detection of the WT gene band after targeting with the T7 system could be due to the non-specific primers used for PCR analysis for those genes (see reference 21). Regrettably, detection of the gene band shared by PKAC1 and PKAC2 after targeting PKAC1 gene with the T7 transient method has been used as an example how an essential gene was determined by PCR analysis (see Fig.2b in reference 21). A similar mistake was made in targeting LmxM.20.1180 gene in reference 20 study (see S3 Fig).

It could be true that if cloning was applied, the null mutants could have been isolated by the T7 transient protocol for some of the five non-essential genes identified by the rRNA-P stable system in this study. However, because two antibiotic repair donors were typically used in the T7 transient method, indeed, null mutants for many non-essential genes could be obtained without cloning if these non-essential genes are present in diploid chromosomes and their deletions have no inhibition effect on the parasite growth such as most of the flagellar protein genes, this has given the impression to Leishmania research community that no cell cloning is needed when using T7 transient CRISPR method other than repeating transfections. Therefore, our advice is that one can continue using T7 transient method without cloning for the first attempt. However, if the null mutant cannot be obtained after the first try, it might be more efficient to clone the transfectants following the second transfection attempt rather than repeating the transfection many times without cloning. If after cloning, a null mutant still cannot be isolated by the T7 transient method, then it is time to consider the stable CRISPR expression approach.

It is important to note that this study was initiated by our curiosity why the deletion mutants for four of the Leishmania flagellar protein genes could not be generated by the T7 transient method. After blast search and sequence analysis, we realized that failure for two of these genes could be due to presence of the conserved sequences in the nearby genes; failure for LmxM.16.1550 (CMF6) could be due to that there are three copies of chromosome 16 in L. mexicana; inability to generate null mutant for LmxM.09.0910 (*Calmodulin*) was likely due to the presence of three copies of this gene in tandem array and its essentiality for Leishmania just like in any other organisms.

**Comment:** A clarification is required about the “stable strategy”: Cells transfected with a plasmid that contains both Cas9 and the gRNA were selected for a period of time. How much time – several days, a week? During this time, gRNA-complexed Cas9 will presumably cut the target locus. Some alleles will be mutated, many will be repaired though homologous recombination with the other allele, but can be cut again. What is the status of the target locus after stable expression of the Cas9-gRNA plasmid, but BEFORE introduction of the donor cassettes? The worry is that non-lethal mutations in the target locus, or reduced gene dosage, could promote adaptation to the loss of gene function. This would facilitate a subsequent full deletion. This is another marked difference in protocol, which makes comparisons between the methods, as presented additionally challenging.

**Response**: Like any stable transfection, it usually takes 10 to 14 days to establish the G418 resistant culture after the promastigotes are transfected with the rRNA-p stable CRISPR expression plasmid (this information has now been added in the methods section see line 408). In other words, 10 to 14 days post the CRISPR plasmid transfection are required to have enough G418 resistant cells for the subsequent antibiotic resistance donor transfection. As the reviewer indicated, Cas9 and gRNA will form the Cas9/gRNA complex soon after Cas9 and gRNA are expressed following the CRISPR plasmid transfection, the Cas9/gRNA complex will scan the genome to identify the specific target site and cleave the target DNA to generate a double-stranded break (DSB) (estimated in less than 1% of cells). Since Leishmania does not have the nonhomologous end-joining (NHEJ) pathway, DSB repair is not efficient in Leishmania. According to our previous studies (references 9,10,12), in the absence of repair donors, more than half of cells with DSB will die due to failure to repair the DSB, remaining DSBs will be repaired through Single Strand Annealing (SSA), Microhomology Mediated End Joining (MMEJ) or Homologous Recombination (HR). DSB repair through SSA or MMEJ will result in deletion of the target DNA sequence (10 to thousands bp). Surprisingly, HR is rarely used in DSB repair in Leishmania (see reference 10). However, in the presence of repair donors, post transfection of the antibiotic resistance marker donor (the donor cassettes) in this study, the majority of DSBs will be repaired by the donor through SSA or MMEJ pathway. Addition of the antibiotic (bleomycin or puromycin) specific to the donor resistance marker will select cells where the DSB is correctly repaired through the donor, the antibiotic will kill the remaining wild type cells and the cells with DSB repaired through SSA or MMEJ but not the donor. Therefore, even if some DSBs are repaired through SSA or MMEJ resulting in target DNA sequence deletions, once the repair donor cassettes are introduced, the majority of DSBs will be repaired through the donor, the subsequent antibiotic selection will eliminate the wild type cells and cells with non-lethal mutations in the target locus.

We understand this reviewer’s concern that stable Cas9 and gRNA expression could increase the chances of unexpected mutations. However, so far no unexpected mutations have been detected through whole genome sequencing of many of the gene disruption and deletion cell lines generated in our lab with the stable rRNA-p CRISPR method.

**Comment:** There is no doubt that different CRISPR protocols have different strengths and weaknesses and they should be used in complimentary ways. Particularly in situations of multi-copy gene families “stable” strategies, such as the one described here, will be invaluable. This is well elaborated in the discussion.

**Response**: We agree. The main objective of this manuscript is to help researchers to better understand the advantages and differences between these two Leishmania CRISPR methods, to apply them more efficiently.

**Comment:** For the data itself, the conclusions form the successful gene deletions with the “stable” method are sound. The conclusions from the unsuccessful attempts are not all that well supported; important controls are missing.

**Response**: We understand this reviewer’s concern. However, in combining the observation of the dying and death of the null mutant clones in a 96 well plate and detection of the wild type gene band in remaining surviving clones, we believe this is sufficient evidence for gene essentiality. The issue of gene essentiality is further addressed in the revised Discussion, lines 335-342.

**Comment:** Figure 1 shows that two genes that did not yield KOs in Ref 21, which used the “transient” method were successfully replaced by the “stable” method. The data showing loss of the WT band is sound.

**Response**: We agree.

**Comment:** Figure 2B. The death of clonal lines is taken as evidence of successful gene deletion. This is perhaps a reasonable assumption, but not proven. Death of the cells does not in itself “indicate all the calmodulin genes [..] had been deleted or disrupted” as stated in line 224. Observing that cells subjected to a CRISPR protocols ended up dead is neither proof of gene deletion (the DNA was not analyzed), nor of gene “essentiality” (ref. Jones et al. 2018). The level of evidence is the same whether this death occurs quickly (in the “transient” protocol) or slowly, as shown here with the “stable” protocol.

**Response**: As our responses to above comments, we believe the dying and death of the calmodulin gene targeting clones resulted from the complete disruption and deletion of the calmodulin gene alleles in those dying clones though we had difficulty in extracting and analyzing the DNA from those dying cells.

We also believe there are differences between the quick death in the transient protocol and the slow death in the stable protocol. The quick death of the cells in the transient protocol could be caused by several reasons (1) the non-transfected wild type cells killed by the two selecting antibiotics; (2) the cells killed by the two selecting antibiotics which were successfully transfected but no gene targeting took place; (3) the partially targeted (only one allele was targeted) cells killed by the second selecting antibiotic; (4) the cells with both alleles targeted by the same antibiotic selection marker donor, those cells would be killed by the another selecting antibiotic; (5) the cells with both alleles successfully targeted by each of the two antibiotic selection marker donors, dying and death of those cells after adding the two selecting antibiotics would be caused by the deletion of the targeting gene if it is essential. Thus, it is very difficult in the transient protocol to distinguish the cause of cell death between failure of the gene targeting and essentiality of a gene in such a large dying cell population.

In contrast, in the stable protocol, only one antibiotic selection marker donor is provided to the parasite. Before cloning into a 96 well plate, the promastigotes are already selected by the antibiotic specific to the selection marker donor, only cells with one or both alleles correctly targeted survive (if the gene is essential, the cells with both alleles disrupted will eventually die, see below). Because the stably expressed Cas9 and gRNA will continue to scan the genome and target the remaining WT gene allele after cloning into a 96 well plate, if the gene is essential for Leishmania viability, once the remaining WT allele in a cloned promastigote is targeted and disrupted, promastigotes in those wells would stop growing or multiply slowly to form clumps until the gene products (mRNA and protein) are diluted and degraded to the minimum level required for survival. Depending on the relative importance, initial abundance, and stability of the gene product in the cell and the cloning time (stage) post the complete gene disruption for an individual clone, the dying (dead) cell clumps could contain only a few cells to more than hundreds of cells (new Fig. 2A and see below). (Note: if the cell dying was caused by the failed double strand DNA break repair, the promastigote would stop proliferating right after cloning and would not be able to form clumps containing more than ten to hundreds of cells; if the cell dying was caused by a spontaneous rare lethal mutation in the genome (unlikely), much fewer dying clones (maybe only one clone) would be observed in a 96 well plate, instead of more than three dying clones regularly observed for disruption of an essential gene). Therefore, the slow dying of the cloned cells in the stable protocol could only result from the complete disruption of the essential gene alleles. In comparison, as described above, multiple factors could cause cell death in the transient protocol.

**Comment:** Figure 2D. The evidence that this clone has both WT and modified calmodulin loci is sound. The conclusion that the genotype is +-- / --- does not follow from these data. The PCR is not quantitative, the ratio of WT vs. modified copies cannot be estimated. Even the possibility that the locus has been amplified (perhaps with a point mutation in the sgRNA targeting site) cannot be excluded. Minimally, the statement “one of the calmodulin genes remains intact” (line 228) should be changed to “at least one…” (as done correctly in line 292). In short, whole genome sequencing of this clone should be done to clarify what the locus looks like in this clone.

**Response**: We agree with the above remarks because for simplicity we omitted the detailed PCR analysis data in Figure 2 in the version submitted. We have now rewritten the text regarding the *Calmodulin* gene targeting and included the detailed PCR analysis data of the *Calmodulin* (+--/---) clone with additional PCR primers in the new Figure (now Figure 3). As you can see in Fig. 3, PCR analysis revealed that CRISPR gene targeting had deleted both alleles of LmxM 09.0930, disrupted both alleles of LmxM 09.0910 and one allele of LmxM 09.0920, and only one wild type LmxM 09.0920 allele remained in this slowest growing clone (Fig. 3B&C). It is interesting to note: a smaller than the expected size (R+10R1) band was detected in this Cal (+--/---) clone, indicating a recombination deletion had occurred in one of the two disrupted LmxM 09.0910 alleles, which explains the additional fainter band running at 700 bp detected in the PCR with (R+L) primer pair. Likewise, the fainter band (the middle band) detected in the PCR with (R+30L1) primer pair could be derived from some of the cells in this Cal (+--/---) clone cell population where recombination deletion took place in the disrupted LmxM 09.0920 allele. Please see the more detailed explanation in Figure 3 legend.

**Comment:** Figure 2D. It is also unclear how stable this genotype is, given the dynamic nature of the Leishmania genome. The quality of the microscopy images in Figs 2C-D is poor, but these round cells look markedly different from the flagellated ones shown in Figure 5B. Additionally, the growth curve in Fig 4 shows they grow at the same rate as WT. This would be surprising given the massive effect on morphology. This cell line could be valuable for studying the functions of calmodulin (outside of the scope of this paper).

**Response**: We agree that this Cal (+--/---) genotype could be non-stable. Indeed, as shown in Fig. 2C (now in Fig. 3E), the promastigotes in the original Cal (+--/---) clone well in the 96 well plate looked very sick, rounded up and small, moved and multiplied slowly (also see the Supplementary move 4). However, likely due to adaptation over time in culture after transferring these cells from the 96 well plate well into the 25 ml flask culture, these Calmodulin mutant promastigotes slowly became more normal in size and proliferation rate, possibly by amplification of the remaining Cal gene. Adaptations in culture is well known in *Leishmania.* Note, the flagellated promastigotes shown in Figure 5B (Now Figure 6B) and the growth curve in Figure 4B (Now Figure 5B) were determined after culturing in the flask for more than five months. Therefore, ideally, it is important to characterize gene mutants soon to avoid adaptation in culture.

**Comment:** Figure 2D. As it is, the presented data lacks sufficient information about the modified gene locus to conclude much, except that this clone is not a null mutant. This is largely the same conclusion that was drawn from the incomplete deletion of this gene by the “transient” method. Whether this failure to remove the gene completely is technical or biological (“essential” gene) cannot be proven by either method, without additional corroborating evidence.

**Response**: As described above, the new Fig 3 and supplementary move 4 clearly show that, (1) only one wild type Calmodulin gene allele (down from six after gene targeting) remained in this slowest growing mutant clone; (2) reduced expression of Calmodulin has fundamental effect on the promastigotes in size, mobility and growth rate; (3) Like in any other organisms, complete disruption of Calmodulin genes is lethal for *Leishmania*.

**Comment:** Figure 3. Knockout of genes on supernumerary chromosomes.
Gene on chromosome 31 – the PCR result looks convincing.
CMF6. All alleles of the gene were lost in some clones following the “stable” protocol. The gene was removed in a minority of the tested clones. This conclusion is supported by the PCR result. The statement that presence of the gene in many clones indicates pressure to retain it is not strongly supported by the evidence. An alternative explanation is that the failure is technical: there could be sequence variation at this locus (perhaps more likely in a trisomic chromosome?) preventing recognition by the gRNA. Or the repair events that happened while cells had the Cas9 plasmid (before addition of the donor DNA) led to small sequence changes that made some alleles refractory to subsequent deletion. The sequence of the remaining allele should be determined to exclude these possibilities. The observation that the null mutants grew slower, is the most convincing evidence supporting the idea that the gene is required for normal growth.

**Response**: We agree that because the CMF6 null mutants grew much slower than the wild type parasites, this suggests that CMF6 is required for normal growth. The statement that the cells have the pressure to retain CMF6 gene should be correct to say, “suggesting there is pressure to retain this gene” (line 250). This is more probable than the sequence variation or mutation in one of the CMF6 alleles prevented the gRNA recognition and targeting. In any event, Fig. 4B provides strong evidence that it was possible to target the CMF6 genes with the stable protocol.

**Comment:** Referring to Line 248 (also 265 ff): The idea that cloning the cells after transfection helps to isolate the null mutant from such mixed populations cases is correct. This is not a new idea and it is equally applicable to slow (“stable”) or fast acting (“transient”) gene deletion protocols. Beneke and Gluenz already published a detailed protocol for clone selection after transfection with the “transient” protocol in 2019 (PMID: 30980304).

**Response**: We agree. Cell cloning was even routinely used to isolate the gene replacement mutant with the traditional homologous recombination gene targeting method. As we mentioned above, in order to isolate the gene null mutants without cloning, the T7 transient protocol decided to use two antibiotic selection marker donors, which is successful for most of the non-essential genes that are diploid and their gene products are not required for the optimal growth of the parasite. Because of the success, people rarely use cell cloning when applying the T7 transient protocol for gene targeting. This is one of the advantages of using T7 transient protocol. However, as demonstrated in this study, no matter which CRISPR method is used, it is necessary to clone the transfectants when targeting the multicopy genes and genes which are required for optimal growth.

**Comment:** Figure 5. The swimming assay in A lacks a negative control that cannot swim. An immobilized WT or a paralyzed or aflagellate mutant could be used to show how passive movement impacts on the recovery of cells from the other side of the tube. These data in themselves may indicate differences in motility, but are not compelling as presented. The results for PKAC1 are however consistent with the data by Fochler et al. Biorxiv 2023 that showed (i) that it was possible to isolate a PKAC1 null mutant also with the “transient” method, and (ii) this mutant was defective in flagellar beating.

**Response**: As suggested, we have now included the formalin fixed WT cells (Dead LmxM WT) as the negative control in the swimming assay in the revised Figure 6. Moreover, this swimming assay is only one of the methods to show the impairment in mobility of these mutant cells as this is also consistent with the supplementary movies revealing altered mobility.

**Other comments:**

The study design is based on the statement that the selected genes had been targeted unsuccessfully for knockout by CRISPR in one of three previous studies (refs 19-21 in the paper). The claim that these were “considered to be essential” is incorrect and should be modified, especially with regards to Ref 19, which did not comment on “essentiality” of any genes.

**Response**: It is true that Refs 19 and 21 (now 20 and 22) did not claim that those genes are essential for Leishmania when their null mutants could not be generated with the T7 transient system. As suggested, we have now removed “considered to be essential” from the text. Instead, we describe these 22 Leishmania genes as below: (See lines 44, 125 and 135 in the revised manuscript): The paper now referrers to these genes as: “22 *Leishmania* genes in which null mutants were not generated using the T7 transient protocol.” “the T7 transient protocol did not generate the live null mutants [20-22]” “in which null mutants were unable to be generated with the T7 transient protocol”.

Note also we have changed the title of the manuscript to: “Evidence for gene essentiality in *Leishmania* using CRISPR”, rather than “Determination of gene essentiality in *Leishmania* using CRISPR.

**Comment: Consideration** should be given to the biological functions of the targeted genes. This information should be added to Table 1.
**Response**: As suggested, we have now added gene function in Table 1.

**Comment:** line 229, “Additional PCR primers (not shown) had been used to verify the gene targeting outcome shown”. Please clarify what this means and include the relevant data or remove the statement.

**Response**: We have now included the additional PCR primers analysis data in new Figure 3.

**Comment:** Line 244, “triploid” means an extra set of chromosomes. Here, the correct term to describe the extra copy of chromosome 16 is “trisomic”.

**Response**: Thank you for correcting this terminology. As suggested, we have revised the sentence as below (see line 247 in the revised manuscript): “the *L. mexicana* LmxM.16.1550 gene (Component of motile flagella 6) (Table 1) is in chromosome 16 which is trisomic in *L. mexicana*.”

**Comment:** Figure 4 please plot growth curves of exponentially growing cells on a semi-logarithmic graph (Y-axis on a log scale). Were doubling times calculated and which differences were significant compared to WT?

**Response**: Depending on the researcher’s preference, we understand that Leishmania growth curves can also be plotted on a semi-logarithmic graph. However, like most Leishmania research labs in the world, we prefer to use the actual cell density (the number of promastigotes /ml) to plot the growth curve, which can more precisely display the Leishmania growth rate and be easier to distinguish the differences between cell lines. As suggested, we have calculated the doubling time for the different cell lines as below:

The doubling time is 8.55 hours for LdWT; 12.29 hours for Ld100590(-);11.3 hours for Ld230540(-);11.3 hours for Ld310120(-);10.21 hours for Ld312380(-). The doubling time differences between LdWT and Ld100590(-), Ld230540(-), Ld310120(-) and Ld312380(-) are statistically significant.

The doubling time is 10.09 hours for LmxWT; 11.74 hours for Lmx343960(-);11.3 hours for Lmx344010(-); 9.69 hours for Lmx201180(-); 12.61 hours for Lmx161550(-); 10.93 hours for Lmx090910(+/-). The doubling time differences between LmxWT and Lmx161550(-) are statistically significant.

In addition, we have now added in the growth curves in Fig.5 the statistical analysis on the cell density differences at day 3 among the different cell lines.

**Comment:** line 304 “its flagellum length appeared normal” – how was this quantified?

**Response**: The relative flagellum lengths of the different *L. mexicana* cell lines were compared on the microscopy photos of the Giemsa-stained promastigotes as shown in Fig. 6B.

**Comment:** Line 439 – “dying crump” - do you mean clump?

**Response**: Thank you. “dying crump” has now been corrected to “dying clump”.

**Comment:** Figure 5. The table in A lacks information. Do the numbers really represent numbers of Leishmania in 3µl (i.e. there were samples that contained 1 parasite)? How many times was each mutant measured? What statistical tests were done to support the statement that some mutants were slower?

**Response**: Yes, the numbers in Fig. 5 now Fig 6, table A represent the true numbers of Leishmania in 3µl in one of the three repeated experiments performed. It is correct, that there were samples that contained none or only 1 promastigote in 3 µl of the right end solution after careful examination under a microscope. As suggested above, we have reperformed the swimming assay and included the formalin fixed WT cells (Dead LmxM WT) as the negative control. This swimming assay was performed a minimum of three times for each of the cell lines, the number differences in the 3µl solution between LmxM WT and PKAC1 null mutant (LmxM.34.4010(-)), CMF6 null mutant (LmxM.16.1550(-)) and the calmodulin partial knockout mutant (LmxM.09.0910(+/-)) are statistically significant by students’ t-test(See new Fig.6: Student’s t-test, ***** P<0.05; ****** P< 0.005; *** P< 0.0005; ******** P< 0.0001).

It is noteworthy that the swimming assay is only one of the methods to show the mobility differences among the mutant cell lines. The supplementary movies further demonstrate the mobility impairment caused by the various disruption mutations.
