# Supplementary material for: Evidence for gene essentiality in Leishmania using CRISPR
Source: PLoS One. 2024 Dec 30;19(12):e0316331. doi: 10.1371/journal.pone.0316331 (PMC11684651; doi:10.1371/journal.pone.0316331)
Supplement: Attachment — Submitted filename: Response to Reviewers.docx [file pone.0316331.s012.docx]

**Response to New additional Comments by Reviewer2**

**Reviewer Comment:** Figure 5. Supports the conclusion that some mutants grow more slowly as promastigotes. (The cell density [cells/ml] should be plotted on a log scale because a semi-logarithmic plot allows for the direct comparison of the growth rates during the exponential growth phase.)

**Response**: As we explained in the previous Response to reviewers, depending on the researcher’s preference, we understand that Leishmania growth curves can also be plotted on a semi-log plot. However, like most Leishmania research labs, we prefer to use the regular plot (the actual cell density; the number of promastigotes /ml) to plot the growth curve. As shown in the supplementary PowerPoint slide (for review purpose only) of comparison between the regular plot and the semi-log plot, it clearly shows that the regular plot can more precisely display the Leishmania growth rate and is easier to distinguish the differences between cell lines.

**Reviewer Comment:** There are still a few sections where clarification would be helpful (some of this information was provided in the extensive response to reviewers – here my suggestions where it would be helpful to add explanations to the manuscript text):

(1) Interpretation of dying cell clumps.

Figure 2. Microscopic observation shows cell clumps indicative of dying cells. The images support the conclusion that these clonal cell lines are not viable. 2B shows PCR products indicating the presence of the wild type LmxM.25.2340 gene as well as the modified gene locus. The interpretation is unclear. If the wild type gene is still detected, these are not KOs. The authors should clarify their interpretation of the PCR results and amend the figure legend as needed. Specifically:
- Did all clones die and form clumps as shown in 2A? Or did some wells still contain live cells that looked different from the clumps?
- Was the PCR in 2B done from wells with clumps (at what time point), or from wells with live cells?
- Do they conclude that one allele is insufficient for survival? Or did they conclude that cells that manage to survive until the final allele is removed. (But for technical reasons DNA can only be analyzed from cells that have not yet reached that final stage).?

There are several other sections where the assumption is made that cells were null mutants but the actual status of the gene locus could/was not determined:

Figure S1. line 647-648. These data do not provide evidence that the gene is essential. Other explanations for the death of the cells cannot be excluded.

For all of these, the only claim that can be made is that the observed decay of the cells is consistent with an essential function of the gene.

**General response to the above reviewer comments (See specific responses below):** We believe we have explained and answered the similar concerns in the previous Response to reviewers. We will explain here in detail once more, which will also answer some of the specific questions. Different from the T7 transient protocol, the gRNA and Cas9 are constantly expressed in the stable CRISPR protocol. Once the CRISPR plasmid pLdCN (pLdsaCN) was transfected into *Leishmania* promastigotes, followed by the antibiotic donor transfection, the gRNA/Cas9 complex would continue scanning the genome until all the WT alleles were targeted and disrupted. If the gene to be targeted is non-essential for viability, the cloned cells would continue to proliferate. Depending on the gene and/or gRNA, PCR analysis of those alive clones will show that either one gene allele or both gene alleles have been disrupted with the antibiotic selection marker, and the smaller WT gene band will not be detected in many of those clones (Fig. 1B). However, if the gene is required for viability and once the remaining copy of the essential gene has been disrupted by CRISPR, cells in those wells would stop growing or multiply slowly to form clumps until the gene products (mRNA and protein) are diluted and degraded to the minimum level required for survival. Depending on the relative importance, initial abundance, and stability of the gene product in the cell and the cloning time (stage) post the complete gene disruption for the individual clone, the dying (dead) cell clumps could contain only a few cells to more than hundreds of cells (Fig. 2A and S1 Fig). Three to ten dying null mutant clones will usually be observed in a 96 well plate when targeting an essential gene. PCR analysis of the remaining surviving clones in the 96 well plate will show the WT gene band persists in all surviving clones and at least one allele of the essential gene was successfully targeted and disrupted by CRISPR (Fig. 2B). In this manner, by combining observation of the death of gene null mutant clones and detection of the WT gene band and the gene targeting band in all surviving clones, using rRNA-P stable CRISPR protocol, we were able to confirm that many of those genes for which the T7 transient protocol was not able to generate alive null mutants (14 out of 22, see table 1, Fig 2, Fig 3 and S1 Fig) are truly essential for *Leishmania* viability.

As suggested by the reviewer, we have revised and added the following sentences into the manuscript text to better explain how the gene essentiality is normally determined with the rRNA-P stable CRISPR protocol:

Line208-210: “To illustrate how gene essentiality is normally determined with the rRNA-P stable protocol, we use targeting AGC essential kinase 1 gene (LmxM.25.2340, AEK1) as an example (Fig 2).”

Line228-233: “In this manner, by combining observation of the death of gene null mutant clones and detection of the WT gene band and the gene targeting band in all surviving clones, using the rRNA-P stable CRISPR protocol, we were able to confirm that many of those genes for which the T7 transient protocol was not able to generate alive null mutants (14 out of 22; see Table 1, Fig. 2, Fig. 3, and S1 Fig.) are truly essential for *Leishmania* viability.”

**Comment:** - Did all clones die and form clumps as shown in 2A? Or did some wells still contain live cells that looked different from the clumps?

**Response:** No, not all clones in the 96 well plate form clumps and eventually die as shown in Fig 2A; most wells in the 96 well plate still contain live cells that looked different from the clumps. These live cell wells are the wells that were used to generate the PCR results in Fig. 2B.

Although the gRNA and Cas9 are constantly expressed in the stable rRNA-P protocol, CRISPR gene targeting is still not very efficient in Leishmania. As described above, depending on the gene and the gRNA activity, 3 to 10 wells with dying null mutant clones(-/-) will usually be observed in a 96 well plate after the double antibiotic (one from pLdCN CRISPR vector, other from the antibiotic resistance repair donor) resistance promastigotes are cloned when targeting an essential Leishmania gene (Fig 2A, Fig 3D and S1 Fig). The live promastigotes (+/-) in the remaining wells will continue proliferation with various growth rate as CRISPR is still targeting the remaining essential gene allele in some of the cells to yield sufficient cells for subsequent PCR analysis. PCR analysis of those surviving clones in the 96 well plate will show the WT gene band persists and at least one allele of the essential gene was successfully disrupted by CRISPR (Fig. 2B and S1 Fig).

**Comment:** Was the PCR in 2B done from wells with clumps (at what time point), or from wells with live cells?

**Response:** The PCR in Fig. 2B (in all PCR analysis in this manuscript) was performed from wells with live cells, which is clearly stated in the text (line 226-228): “PCR analysis of all the surviving clones in the 96 well plate will show the WT gene band persists and at least one allele of the essential gene was successfully targeted and disrupted by CRISPR (Fig 2B, S1 Fig and below)”.

**Comment:** Do they conclude that one allele is insufficient for survival? Or did they conclude that cells that manage to survive until the final allele is removed. (But for technical reasons DNA can only be analyzed from cells that have not yet reached that final stage).?

**Response:** As described above and demonstrated in this manuscript, we conclude that one essential gene allele is sufficient for Leishmania parasite survival.

**Comment:** There are several other sections where the assumption is made that cells were null mutants but the actual status of the gene locus could/was not determined:

Figure S1. line 647-648. These data do not provide evidence that the gene is essential. Other explanations for the death of the cells cannot be excluded.

For all of these, the only claim that can be made is that the observed decay of the cells is consistent with an essential function of the gene.

**Response:** As we explained in our previous Response to reviewers, we had difficulty extracting the genomic DNA from the dying cell clumps for PCR analysis. However, we believe the disruption of all wild type essential gene alleles in those clones is the most likely cause of the cell dying and death as explained and illustrated in the section of essential genes (line 206-282) in the text using the AGC essential kinase 1 gene (LmxM.25.2340, AEK1) as a typical example (Fig. 2) to show how gene essentiality is normally determined with the rRNA-P stable protocol.

In addition, if the cell dying after cloning the double antibiotic resistance promastigotes into a 96 well plate was caused by the failed double strand DNA break repair, the promastigote would stop proliferating right after cloning and would not be able to form clumps containing more than ten to hundreds of cells; if the cell dying was caused by a spontaneous rare lethal mutation in the genome (unlikely), much fewer dying clones (maybe only one clone if it did happen) would be observed in a 96 well plate, instead of more than three dying clones regularly observed for disruption of an essential gene.

We understand the concern raised by this reviewer that despite the complete disruption of the essential gene alleles by the stable CRISPR gene targeting protocol is the most likely cause of cell death observed in the 96 well plate, due to difficulty to extract genomic DNA from the dying cell clumps, we did not have direct evidence so far to show that both the essential gene alleles in those dying promastigotes clumps were indeed disrupted by CRISPR with the antibiotic selection marker donor. However, it is important to point out that the DiCre inducibe gene deletion method, the most stringent method before CRISPR and recommended by this reviewer (see reference 2), also relies on observation of the cell death after complete deletion of the essential gene alleles to determine Leishmania gene essentiality. DiCre method also did not provide direct evidence (or definitive proof) to show that the floxed remaining essential gene allele in those dying Leishmania cells was indeed excised by the DiCre after addition of rapamycin.

To identify potential new drug targets, it is necessary to clearly determine the essentiality for all *Leishmania* genes [2]. The T7 transient system currently employs two methods to determine whether a gene is essential for *Leishmania* viability. Method 1 is to see, like the traditional gene targeting, if it is possible to delete the two chromosomal copies of the essential gene of interest by providing the cell with an extrachromosomal copy of the gene [2, 21,22]; Method 2 is to repeat the targeting attempt for a minimum of three times, if the null gene mutant could still not be generated with T7 system after three attempts, the gene can be considered as essential for *Leishmania* [21].Thus, both methods are labor intensive and time consuming. Method 2 at times could be not reliable as 4 to 6 attempts with T7 system were sometimes required to generate the null mutants for non-essential genes as shown in the *L. donovani* membrane protein gene study [22]. Nevertheless, as demonstrated in this study, using the rRNA-P stable protocol with single CRISPR plasmid and single antibiotic selection marker donor transfection, it was possible to determine gene essentiality by combining observation of the death of gene null mutant clones and detection of the WT essential gene band and the gene targeting band in all surviving clones in a 96 well plate [9,10,12,14].

**Comment:** line 45 (and 138-140) – “by directly observing gene null mutant promastigotes dying in culture”. The observation is: dying promastigotes. When these genes are targeted with the transient protocol, dying promastigotes are also observed. Whether or not the targeted gene is absent in these promastigotes is not known, in either case. All that can be concluded (in both protocols) is that failure to recover live cells is consistent with an essential function of the targeted gene (but not definitive proof).

**Response:** Although the complete sentence in line 43 should be “The rRNA-P stable protocol provides evidence for gene essentiality by directly observing null mutant promastigotes dying after cloning the antibiotic resistance promastigotes into a 96 well plate”, we believe line 43 (and 127-128) – “by directly observing gene null mutant promastigotes dying in culture” is appropriate in Abstract and Introduction because the Results section text will explain what it means the dying null promastigotes in culture after cloning into a 96 well plate.

As we explained in our previous Response to reviewers, the dying promastigotes observed in transient protocol could be caused by several reasons (1) the non-transfected wild type cells killed by the two selecting antibiotics; (2) the cells killed by the two selecting antibiotics which were successfully transfected but no gene targeting took place; (3) the partially targeted (only one allele was targeted) cells killed by the second selecting antibiotic; (4) the cells with both alleles targeted by the same antibiotic selection marker donor, those cells would be killed by the another selecting antibiotic; (5) the cells with both alleles successfully targeted by each of the two antibiotic selection marker donors, dying and death of those cells after adding the two selecting antibiotics would be caused by the deletion of the targeting gene if it is essential. Thus, it is very difficult in the transient protocol to distinguish the cause of cell death between failure of the gene targeting and essentiality of a gene in such a large dying cell population.

In contrast, in the stable protocol, only one antibiotic selection marker donor is provided to the parasite. Before cloning into a 96 well plate, the promastigotes are already selected by the antibiotic specific to the selection marker donor, only cells with one or both alleles correctly targeted survive (if the gene is essential, the cells with both alleles disrupted will eventually die, see below). Because the stably expressed Cas9 and gRNA will continue to scan the genome and target the remaining WT gene allele after cloning into a 96 well plate, if the gene is essential for Leishmania viability, once the remaining WT allele in a cloned promastigote is targeted and disrupted, promastigotes in those wells would stop growing or multiply slowly to form clumps until the gene products (mRNA and protein) are diluted and degraded to the minimum level required for survival. Therefore, the slow dying of the cloned cells in the stable protocol could only result from the complete disruption of the essential gene alleles. In comparison, as described above, multiple factors could cause cell death in the transient protocol.

**Comment:** Figure S1. line 647-648. “Morphology of clumping dying clones (-/-) once the remaining copy of the gene has been disrupted by CRISPR.” The PCR shows that the WT band was present in all samples. No evidence is presented to show that the remaining copy of the gene has been disrupted. It is possible that this is what happens, likely even in many cases, but the data does not establish a direct link between the loss of the gene and the death of the cells. If the authors’ interpretation was correct, there should be a decrease of the WT PCR product over time. There is no evidence that the WT band decreases with time.

**Response:** As we explain it in the manuscript, in the previous Response to reviewers and in the above responses to reviewers, in the rRNA-P stable CRISPR protocol, gene essentiality is determined by combining observation of the death of gene null mutant clones and detection of the WT gene band and the gene targeting band in all surviving clones.

Nevertheless, we have revised the legend for S1 Fig (see line 618-644) to more clearly indicate that both the WT essential gene band and the gene disruption band were detected in all surviving clones (+/-) tested in the 96 well plates, and the observation of the dying and death promastigotes clumps of the essential gene null mutants’ clones (-/-) in the 96 well plates.

Because the gRNA/Cas9 complex will continue targeting the gene of interest in the rRNA-P stable protocol, as the reviewer suggested, we believe that depending on the gRNA activity (that is if the CRISPR gene targeting rate is faster than the cell proliferation rate), it could be true that the WT PCR product in the gene targeting population would be reduced over time, which, however, needs to be verified by quantitative PCR analysis. This is beyond the scope of this study.

**Comment:** Note, Figure S1 legend. Lines 650-653 are a duplication of lines 224-227 in the main text.

**Response:** We have revised S1 Fig legend to avoid duplication of lines 224 -227.

**Comment:** (2) Ability to delete protein kinase A
Table 1. Null mutants for LmxM.34.3960 and LmxM.34.4010 (Protein kinase A catalytic subunit isoforms) were not confirmed in the cited reference but they were subsequently reported in Fochler et al., Biorxiv 2023 (for completeness this could be added to footnote 6). This successful KO also used the transient protocol; it is likely it was able to confirm the KO because gene-specific primers were used, unlike the reference cited in the manuscript.

**Response:** We have now included this new study by Fochler et al in the reference list and added this new information to footnote 6 of Table 1 as below and in the discussion (see line 399-402).

6. Fochler et al have recently shown that LmxM.34.3960 and LmxM.34.4010 live null mutants could also be generated with T7 transient protocol when the gene-specific primers were used to confirm the CRISPR deletion mutants [26].

26. Fochler S, Walker BJ, Wheeler RJ, Gluenz E. Divergent Protein Kinase A contributes to the regulation of flagellar waveform of the motile flagellum of *Leishmania mexicana.* bioRxiv 2023.11.08.566240; doi: https://doi.org/10.1101/2023.11.08.566240

**Comment:** (3) Claims about calmodulin
Figure 3B, C, line 253-254. I do not follow the argument that “only one wild type LmxM.09.0920 allele remained”. There are 3 PCR bands in the mutant – it would be helpful to label them in the illustration (WT, modified locus, ...?).
How was allele copy number determined?

**Response:** As in our previous Response to reviewers, we explain again here and in the revised Fig 3 legend (Line 260-282) that PCR analysis revealed only one wild type *calmodulin* gene allele (LmxM.09.0920 allele) remained in this slowest growing *Calmodulin* gene targeting clone. *Leishmania* contains 3 *calmodulin* genes (LmxM.09.0910, LmxM.09.0920 and LmxM.09.0930) in tandem array in diploid chromosome 9 (Fig 3A), therefore, there are total of six copies of *calmodulin* gene (+++/+++). After CRISPR gene targeting, the PCR product size with primers 10R1+30L1 reduced from 4.2 Kb of the WT band size to 2.8 Kb and no PCR product could be detected with primers 30R1+L, indicating both LmxM.09.0930 alleles had been deleted (++-/++-). The PCR product sizes of primers R+10 L1 increased from 700 bp to 900 bp and 1100 bp respectively, indicating both LmxM.09.0910 alleles had been disrupted (-+-/-+-). Both the WT band and size increased disruption band of primers R+30L1 were detected, indicating one LmxM.09.0920 allele had been successfully disrupted and another WT LmxM.09.0920 allele (the only WT *Calmodulin* gene allele) remained in this clone (---/-+-). Note: a smaller than the expected size (R+10R1) band (900 bp) was detected in this Cal (+--/--- = ---/-+-) clone, indicating a recombination deletion had occurred in one of the two disrupted LmxM 09.0910 alleles, which explains the additional fainter band running at 700 bp detected in the PCR with (R+L) primer pair. Likewise, the fainter band (the middle band) detected in the PCR with (R+30L1) primer pair could be derived from some of the cells in this Cal (+--/---) clone cell population where recombination deletion took place in the disrupted LmxM 09.0920 allele. Briefly, since the WT *Calmodulin* gene band was detected in all the surviving clones together with the observation of dying null mutant clones (Fig. 3 and S1 Fig), this strongly indicates that like in other organisms, Calmodulin is essential for *Leishmania*.

**Comment:** Line 250 “data not shown” – please show data or remove statement.
**Response:** We have now included the data in S1 Fig.

**Comment:** Line 257 “Calmodulin is only one of the 98 Leishmania flagellar protein genes” required for viability. It isn't clear if this means “only one of many” or “the only one”.

**Response:** As suggested, we have now revised the sentence as below: “*Calmodulin* is the only one of the 98 *Leishmania* flagellar protein genes which is required for *Leishmania* viability.”
